# Temporal variation of human encounters and the number of locations in which they occur: a longitudinal study of Hong Kong residents

**DOI:** 10.1098/rsif.2017.0838

**Published:** 2018-01-24

**Authors:** Kin On Kwok, Ben Cowling, Vivian Wei, Steven Riley, Jonathan M. Read

**Affiliations:** 1 The Jockey Club School of Public Health and Primary Care, The Chinese University of Hong Kong, Hong Kong Special Administrative Region, People's Republic of China; 2 Stanley Ho Centre for Emerging Infectious Diseases, The Chinese University of Hong Kong, Shatin, Hong Kong, Hong Kong Special Administrative Region, People's Republic of China; 3 Shenzhen Research Institute, Chinese University of Hong Kong, Shenzhen, People's Republic of China; 4 WHO Collaborating Centre for Infectious Disease Epidemiology and Control, School of Public Health, Li Ka Shing Faculty of Medicine, The University of Hong Kong, Hong Kong Special Administrative Region, People's Republic of China; 5 MRC Centre for Outbreak Analysis and Modelling, Department for Infectious Disease Epidemiology, Imperial College, London, UK; 6 Centre for Health Informatics, Computation and Statistics, Lancaster Medical School, Faculty of Health and Medicine, Lancaster University, Lancashire, UK; 7 Institute of Infection and Global Health, The Farr Institute@HeRC, University of Liverpool, Liverpool, UK

**Keywords:** social contact, longitudinal, temporal variations

## Abstract

Patterns of social contact between individuals are important for the transmission of many pathogens and shaping patterns of immunity at the population scale. To refine our understanding of how human social behaviour may change over time, we conducted a longitudinal study of Hong Kong residents. We recorded the social contact patterns for 1450 individuals, up to four times each between May 2012 and September 2013. We found individuals made contact with an average of 12.5 people within 2.9 geographical locations, and spent an average estimated total duration of 9.1 h in contact with others during a day. Distributions of the number of contacts and locations in which contacts were made were not significantly different between study waves. Encounters were assortative by age, and the age mixing pattern was broadly consistent across study waves. Fitting regression models, we examined the association of contact rates (number of contacts, total duration of contact, number of locations) with covariates and calculated the inter- and intra-participant variation in contact rates. Participant age was significantly associated with the number of contacts made, the total duration of contact and the number of locations in which contact occurred, with children and parental-age adults having the highest rates of contact. The number of contacts and contact duration increased with the number of contact locations. Intra-individual variation in contact rate was consistently greater than inter-individual variation. Despite substantial individual-level variation, remarkable consistency was observed in contact mixing at the population scale. This suggests that aggregate measures of mixing behaviour derived from cross-sectional information may be appropriate for population-scale modelling purposes, and that if more detailed models of social interactions are required for improved public health modelling, further studies are needed to understand the social processes driving intra-individual variation.

## 1. Introduction

The transmission of acute respiratory infections is thought to be driven by multiple factors, including the rate of social interactions and the duration of exposure [1]. In general, individuals who have high connectivity are considered to be at elevated risk of infection and of passing infection on [2], and control interventions which target those individuals are often efficient, particularly for sexually transmitted infections. It is an open question as to whether such an approach is feasible for respiratory infections, and the link between social connectivity and infection risk for respiratory infection has only recently received research attention [3,4].

Representative studies which quantify patterns of social encounters are few, and are typically limited to the characterization of social mixing behaviour of individuals over a single day [3]. A few smaller studies have measured encounter patterns of individuals over multiple days, but are generally limited to 2 day samples, and have focused on quantifying differences between school-term and holidays for schoolchildren [5], and contrasting days of wellness and illness [6]. Longer studies of encounter patterns have so far been conducted in small and potentially non-representative sample [7–9]. There is, therefore, a need to understand how stable or consistent the mixing behaviour of individuals is over longer periods of time, for determining both the reliability of information gained from single day studies and for the ability to identify and target individuals at high risk of infection. The most appropriate measure of contact rate is also unclear. Both the number of different individuals encountered and the time spent with them are important for transmission, but it is unclear how they may combine with exposure to infectious individuals to generate infection risk. Consequently, several studies of contact mixing patterns report the total number of contacts and estimate the total contact duration [10,11].

Between-individual variation in the rate at which contact occurs is known to have important implications for the transmission of infectious diseases and its control [12]. Daily differences in the behaviour of an individual can also impact transmission, particularly if triggered by illness [13,14]. For many acute infectious diseases that are spread through close contact, infectious individuals can pose a transmission risk for several consecutive days until the infection is cleared or treated. This may be particularly important for influenza, where individuals may be infectious prior to symptoms developing [15]. The set of people such individuals may encounter during this infectious period defines their effective neighbourhood of contacts—the totality of people they could potentially infect [16]. In other words, the speed and extent to which infection can transmit may be determined by how quickly contacts are made and how the number of people encountered may accumulate during the infectious period. The number of different people encountered by an individual may asymptote as the number of days considered increases [7]. This saturating relationship may reduce the final variation between individuals' effective neighbourhood size, such that variation in the number of secondary infections arising may not be as great as estimated by information from a single day, particularly for infections with long (multiple day) infectious periods. Currently, there is little evidence as to how individuals’ contact rates may change over time [3,7,17]. Understanding how effective neighbourhood size may vary in different populations and for different infections has import implications for public health control, including the effort that should be invested in contact tracing during outbreaks.

Hong Kong is a densely populated city of more than seven million residents; it is an important city for international travel, with strong regional and international communication links. This connectivity is reflected in its significance for infectious diseases: SARS emerged in the region and spread to the rest of the world through Hong Kong [18]. Annual seasonal influenza is also thought to originate in the region [19]. A previous survey of social mixing behaviour was conducted in Hong Kong to examine how social connectivity related to incidence of influenza infection during the 2009 pandemic influenza [20,21]. Here, we present an extension of that work: a longitudinal study of the social mixing behaviour of Hong Kong residents, where participants reported on the social encounters they made on up to four different days over 17 months. Using the information collected by the study, we explore the patterns and variation in three key contact rates—the number of people encountered (number of contacts), the total duration of contact events and the number of different locations in which contacts are reported.

## 2. Methods

### 2.1. Study overview

**Figure 1.**
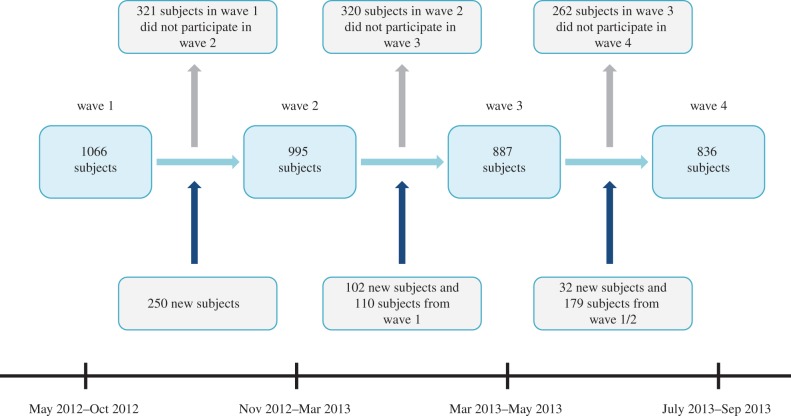
Timeline of the study, showing the four waves of study participation. The duration of study was 17 months.

We followed an open cohort of individuals belonging to recruited households, over 17 months between May 2012 and September 2013. Four waves of telephone interviews were arranged to start in May 2012, November 2012, March 2013 and July 2013, with the duration of each recruitment period lasting between three and six months. The timing of study waves was as follows: wave one (R1) ran from May 2012 to October 2012; wave two (R2) from November 2012 to March 2013; wave three (R3) from March 2013 to May 2013; wave four (R4) from July 2013 to September 2013 (figure 1). Questionnaires (contact diaries)—soliciting information on social encounters made during a randomly assigned day—were administered to participants in each wave via a telephone interview. Contact diary information was collected from each participant for up to four different days (one in each wave of the study). Contact information recorded the number of distinct individuals encountered, the duration of contact events with each and the number of distinct locations in which contact occurred.

### 2.2. Recruitment

Households were the main recruitment unit for this study. In the early stage of the study (May 2012), a telephone recruitment company was commissioned to recruit all study households. We aimed to recruit approximately 1000 households. Households participating in an existing cohort study [22] were invited to participate in this study; additional households were also recruited by random dialling digit using the sampling framework used to recruit households into the existing cohort [22]. Both recruitment arms solicited participating households from the Hong Kong population, and all households were initially identified and approached via random-digit dialling and an initial telephone call to a fixed-line number. All individuals who typically slept in the household for at least five nights per week were eligible to enter the study; domestic helpers were ineligible for study participation due to concerns regarding coercion. The minimum age for participation was 2 years old; there was no upper age limit; all eligible members of the households were invited to have four telephone interviews. Additional households were recruited as required during each study wave to balance losses to follow-up.

### 2.3. Reporting contacts

Participating households received a study booklet at the start of their participation describing the purpose of the study, their involvement, the definition of contact and examples of the types of contact the study will ask them to report. Contact was defined as a social encounter with an individual which included a face-to-face conversation or touch (such as handshake, a kiss, games and sports or similar events involving body touch). For each study wave, participating households were assigned a date for which their contact behaviour was to be reported (hereon referred to as the *reporting day*). They would be interviewed about this reporting day within 4 days after the reporting day (referred to as the *interview date*). All individuals within a recruited household were assigned the same interview date and reporting day within each wave. The reporting day was allocated sequentially within the study wave period. The household was contacted and informed of their reporting day and interview date, with both dates being reallocated later in the wave and the process repeated if the participants communicated that they were unavailable for interview on the first interview date. Following the reporting day, households were contacted by the study team on the interview date, and the team administered a questionnaire (also called a contact dairy) on each eligible participant within that household to collect recalled information on their contact behaviour during the reporting day.

Participants were asked to recall all contact events—defined as encounters with distinct individual or group of individuals in a particular geographical location—during a reporting day [10]. The number of individuals associated with a contact event could be reported and recorded as either single individuals, or as groups of individuals sharing the same attributes within the same contact event [10,11]. For a participant's reporting day, interviewers recorded all contact events reported by the participant, a name or description of the contact or group associated with that event, and a name or description of the geographical location in which it occurred, to distinguish between different locations during the interview. Additionally, for each contact event, they also recorded the number of individuals within a group contact, the duration, age, social setting (home, school, work, other), whether the encounter included touch, and the typical frequency the participant would encounter that contact. The number of unique people with whom a participant reported contact during an interview (hereon referred to as number of contacts) was defined as the number of unique contact descriptors associated with each contact event multiplied by the number of individuals represented by the contact event. Descriptors of contacts were anonymized, and did not identify people in such a way as to identify a repeat encounter with a contact by the same participant (across study waves) or encounters with the same person by two or more participants. Additional information on the recording of contacts and locations by the study is provided in the electronic supplementary material (appendix A).

Interviewers reviewed the contact event information and confirmed the information with the participant where multiple contacts had the same or similar names or descriptions. In turn, eligible participants in the household were interviewed. The above procedure was repeated in each wave of recruitment. Participants who agreed to complete the questionnaire were compensated with HKD20 of supermarket vouchers for each interview in which they participated. Individuals were permitted to participate in subsequent waves even if they missed one or two waves.

### 2.4. Age mixing matrices

To describe the pattern of social mixing and quantify the tendency of people to mix with others of similar ages or different ages over time, we calculated age-based mixing matrices of participants in four waves of recruitment with four groups of participant ages (5–19, 20–39, 40–64, 65+) and five groups of contact ages (0–5, 6–19, 20–39, 40–64, 65+) based on the ratio of the measured probability of a contact between individuals under an assumption of proportionate mixing [10]. We excluded information from participants in the two to four age group due to small sample sizes. Proportionate mixing was calculated using the age distribution from the 2011 Hong Kong census [23]. Ratio values above one in the matrix indicate more contact than expected at random between the pair of age groups, and values below one indicate less contact than expected. Confidence intervals were calculated by 1000 bootstrap resampling of participants.

### 2.5. Estimation of total contact duration

While the number of individuals a participant may encounter can be a useful measure of their social connectivity, from the perspective of infection by respiratory pathogens, the duration for which they may be exposed to pathogens via social interactions may be just as important. Following established methodology [10,11], we estimated the total duration each participant was in contact with other people during a reporting day. Firstly, we fitted an exponential model to the observed distribution of categorized durations recorded for all contacts using an adaptation of the expectation–maximization algorithm. Secondly, drawing randomly from this model, we assigned durations (minutes) to each contact event reported. Thirdly, we repeated this process 200 times, to permit estimation of uncertainty in derived duration metrics. Finally, the total contact duration was found by summing the estimated contact durations for each contact event, for each participant for each day they reported. We assumed interaction with groups (more than one person) to contribute towards the total contact duration as would a contact event with a single individual.

### 2.6. Statistical analysis

To derive overall averages of number of contacts, duration of contact and number of locations, we calculated the mean of participants' means to account for repeated observations per participant. We explored the variation of the accumulation of contacts over multiple days using only participants who reported contact information from all four reporting days.

We applied multivariate mixed-effect regression models to the data using total number of contacts, total duration of contact events and number of locations in which contacts were encountered as response variables. Specifically, log(1 + *K_ij_*), log(1 + *D_ij_*) and log(1 + *L_ij_*) are defined as the response variables with Gaussian distributions, where *K_ij_* is the total number of contacts reported by participant *i* during survey wave *j*, *D_ij_* and *L_ij_* are the equivalent variables for total duration of contact events and number of locations. Model fitting was performed using information from participants with two or more observations and implemented within the *R* statistical language [24] using the *gamm4* package [25]. Random effects were modelled as participant-specific intercepts. Explanatory variables included age at interview date and sex, study wave (R1 to R4, to test for temporal effects), and the day of reporting (to understand the effect of different days of the week). For models with number of contacts and total duration as response variables, we also included the number of contact locations reported (categorized as 0, 1, 2, 3, 4, 5 and 6 or more) as an additional explanatory variable, to understand the contribution of multiple locations to contact rates. We fitted penalized thin-plate splines to explore the potential for nonlinear relationships of the explanatory variables with age at interview date in decimal years (i.e. measuring age as number of months). Percentage contributions for each of the covariates were calculated by predicting the relevant contact rate as a percentage of the predicted modelled rate for a comparator set of covariate values; we used a 50-year-old male, reporting contacts on a Monday in the first study wave, with a household size of one and with a single contact location as the comparator. Additional supporting regression models were fitted for alternative response variables and exploratory variables: these models are described within the electronic supplementary material (appendices F and H).

## 3. Results

### 3.1. Sample size and demography

Overall, 1450 individuals from 857 households were recruited, of whom 401 took part in all four waves of recruitment, 402 took part in exactly three waves, and 327 and 320 individuals took part in exactly two and one wave, respectively. Across 4 study waves, 3784 interviews were conducted, 98.5% of which were successfully made within 4 days of the reporting day. Thirty per cent of the participants taking part in the current study wave did not participate in the subsequent wave: 321 subjects out of 1066 participating in wave 1 did not take part in wave 2; 320 out of 995 subjects in wave 2 did not participate in wave 3; 262 out of 887 participating in wave 3 did not participate in wave 4. Recruitment of additional participants and repeated follow-up of previously participating individuals helped to maintain a large number of subjects across four waves of recruitment (figure 1).

Twenty-six participants did not provide complete personal demographic information (such as age) or contact information: these subjects were excluded from all analyses requiring the missing information. We found no difference in the age distribution and sex of participants between study waves, though there was a difference between study waves in the days of the week for which contacts were reported (table 1). There was no statistical difference between the distribution of participants in terms of age or sex across the four waves of recruitment, though there was difference between waves in the distribution of weekdays recorded by participants (table 1). Children were underrepresented in our sample, while adults and females were overrepresented (electronic supplementary material, figure S1).

**Table 1.** Characteristics of the study subjects.

	number of participants (%)				
	wave 1 *N* = 1066	wave 2 *N* = 995	wave 3 *N* = 887	wave 4 *N* = 836	*p*-value^b^
age					
2–4	1 (0.1)	1 (0.1)	0 (0.0)	1 (0.1)	0.571
5–19	80 (7.5)	61 (6.1)	56 (6.3)	43 (5.1)	
20–39	203 (19.0)	189 (19.0)	182 (20.5)	166 (19.9)	
40–64	629 (59.0)	586 (58.9)	501 (56.5)	480 (57.4)	
65+	144 (13.5)	145 (14.6)	138 (15.6)	141 (16.9)	
not recorded	9 (0.8)	13 (1.3)	10 (1.1)	5 (0.6)	
sex					
male	416 (38.0)	393 (39.5)	347 (39.1)	332 (39.7)	0.990
female	650 (61.0)	602 (60.5)	540 (60.9)	504 (60.3)	
not recorded	0 (0.0)	0 (0.0)	0 (0.0)	0 (0.0)	
weekday					
Sunday	154 (14.4)	123 (12.4)	133 (15.0)	160 (19.1)	<0.001
Monday	196 (18.4)	116 (11.7)	195 (22.0)	132 (15.9)	
Tuesday	163 (15.3)	241 (24.2)	98 (11.0)	97 (11.6)	
Wednesday	125 (11.7)	133 (13.3)	90 (10.1)	83 (9.9)	
Thursday	140 (13.1)	131 (13.2)	119 (13.4)	97 (11.6)	
Friday	159 (14.9)	87 (8.7)	114 (13.0)	109 (13.0)	
Saturday	129 (12.1)	164 (16.4)	137 (15.4)	157 (18.8)	
not recorded	0 (0.0)	0 (0.0)	0 (0.0)	0 (0.0)	
response rate (%)^a^	74.9	69.9	62.3	58.7	

^a^Calculated based on 1424 participants who had ever participated in any waves of the recruitment with full information of age, sex and weekday.

^b^Chi-square test for independence.

### 3.2. Distribution of contact rates and number of locations where contact occurred

**Figure 2.**
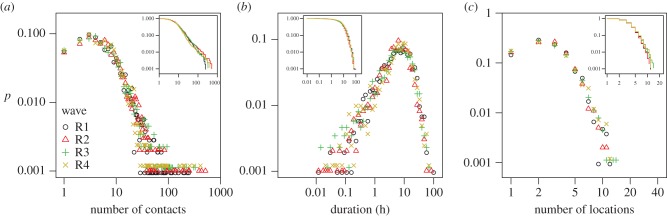
Normalized distributions of (*a*) the number of contacts and (*b*) the total duration of contact events made, and (*c*) the number of locations at which contact events occurred for each of four waves of sampling. Waves are represented by unique colours and symbols as shown in *a*. Durations were binned into log-distributed periods prior to plotting. Inset plots show the corresponding inverse cumulative probability distributions for each wave, colour coded as for the main plots.

We found a remarkable consistency in the overall distribution of number of contacts reported by participants between waves (figure 2*a*), with each wave having comparable mean values (electronic supplementary material, table S2) and showing a similar long-tailed degree distribution of contacts (figure 2). The pattern of this distribution, particularly the long right-hand tail, was similar to the distribution observed in similar studies in China [10] and the UK [11,26]—studies which also were designed to enable participants to report large numbers of contacts easily by reporting groups of similar contacts. We also found distribution consistency between waves for both total contact duration and number of locations (figure 2*b*,*c*). Chi-squared tests showed no significant difference in the distributions between waves of the number of contacts or the number of locations reported; however, distributions of duration were different between waves (*p* < 0.001). Stratified by study wave, the number of contacts made by age groups of participants also showed a similar pattern (electronic supplementary material, figure S3). Across all four waves of study, the mean average daily number of contacts reported was 12.5, recorded in an average of 2.9 different locations, while the mean duration of contact events was 9.1 h per day.

While the aggregate distribution of number of contacts was very similar between waves, we found considerable variation at the individual level (electronic supplementary material, table S3 and appendix G). The distribution of the difference between number of contacts made by a participant in any two waves was similar (electronic supplementary material, figure S2A), and there was a slight negative correlation between the number of contacts reported in any two waves (ranging from −0.034 to −0.005, but not significant), though a positive correction between waves 3 and 4 (electronic supplementary material, table S3). Among all possible pairs of wave comparison, only duration of contacts between wave 2 and 3 was found to be significantly correlated though the strength of the correlation was weakly positively (electronic supplementary material, table S3). Individual-level variation between waves was also observed for total contact duration (electronic supplementary material, figure S2B). For the number of locations in which contact occurred, again there was variation at the individual level (electronic supplementary material, table S3, figure S2C). We found a weak positive correlation between individual participant's coefficients of variation for number of contacts, contact duration and number of locations (electronic supplementary material, figure S12).

### 3.3. Patterns of mixing between age groups

**Figure 3.**
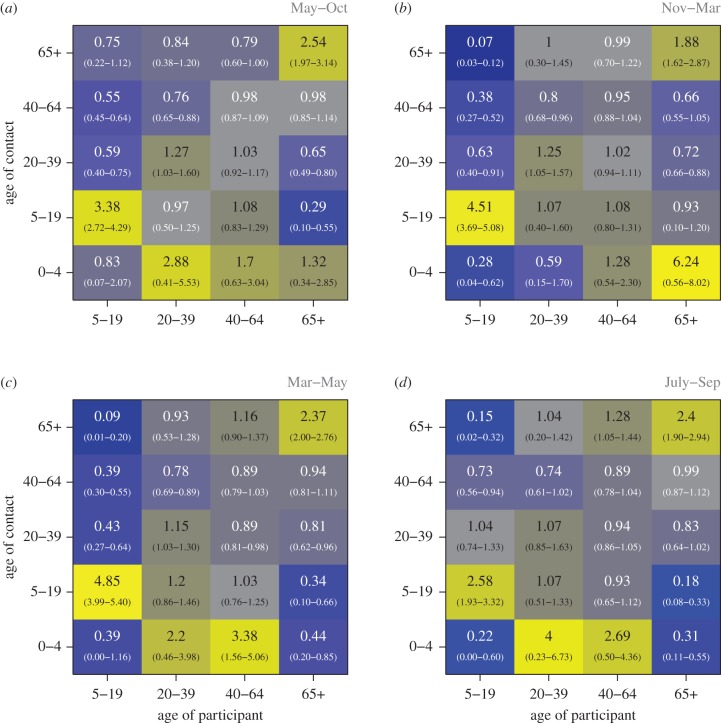
Age mixing matrices, stratified by subsequent study waves (R1, R2, R3, R4, *a*–*d*, respectively). Bluer colours indicate less mixing between age groups than expected by random mixing, and yellower colours indicate more mixing. 95% confidence intervals are shown in the parenthesis, derived from 1000 re-samples of participant contact diaries.

The manner in which age groups interact with their own and other age groups is important for the spread of infection within a population [27]. We found broadly similar patterns of mixing between age categories across the four study waves (figure 3; electronic supplementary material, table S4 and figures S3 and S4), though there are some differences which may be important when considering the potential spread of infections. All age groups, except the 20–39 and 40–64 groups in wave 4, were significantly more likely to have a greater number of contacts with a member of their own age group than would be expected if mixing were at random across all four waves: this is indicative of age-assortative mixing. The strongest assortative mixing rates were made by younger (5–19 years old) and older (65+) participants: these individuals were, respectively, at least 3.4 and 1.9 times as likely to have contact with individuals of their own age than would be expected by proportionate mixing, in study waves 1 to 3 (figure 3). In comparison, wave 4 showed reduced assortative mixing of the younger age group (5–19 years old). This may be due to more sampled days within this wave coinciding with the summer school holidays than for other waves. This explanation is supported by an observed reduction in the average number of contacts made in school by this age group in wave 4 (electronic supplementary material, table S5). From an infectious disease perspective, wave-to-wave differences in assortative mixing do translate into differences in epidemic growth rates, with wave 2 having the fastest growth (electronic supplementary material, figure S5, appendix E). Similar average aggregate age mixing patterns were observed for skin-on-skin touch contacts (electronic supplementary material, table S6), which may be a more appropriate representation of a transmission opportunity for particular diseases [7,28].

### 3.4. Association of contact rates with demographic variables, study wave and weekday

To assess the variation in contact behaviour at the individual level, while adjusting for factors thought *a priori* to be associated with contact rate, we fitted mixed effect regression models to the contact metrics. We modelled the effect of participant age and sex, day of the week, number of locations in which contact was reported (if included), and study wave on the total number of contacts reported by participants, estimated total contact duration and the number of locations visited where contact occurred as independently fitted models. All models accounted for repeated observations from participants.

**Figure 4.**
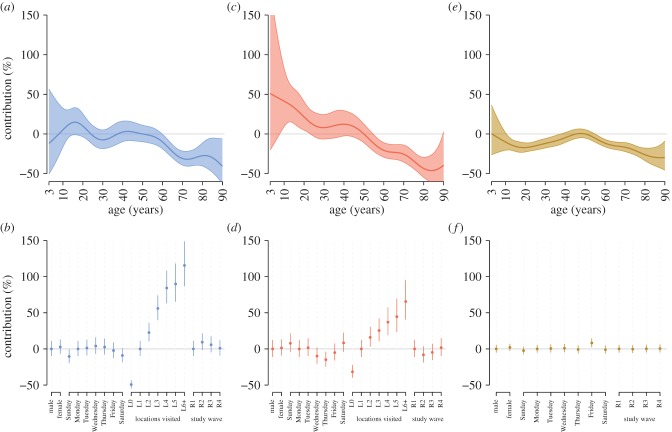
Estimates of percentage contribution in the predicted number of contacts (*a* and *b*), the total duration of contact events (*c* and *d*) and the number of locations in which contact occurred (*e* and *f*) from the regression models for different characteristics of the participants relative to a 50-year-old male taking part in study wave 1, making contacts in 1 location on a Monday. (*a*,*c*,*f*) The splines fitted to age for the two models; (*b*,*d*,*e*) the percentage contribution for sex, day of the week, categorized number of locations in which contact occurred (L0 to L6+) if included, and study wave (R1 to R4). 95% confidence intervals are denoted by a shaded region (*a*,*c*,*e*) or vertical line (*b*,*d*,*f*).

We found a significant nonlinear association between the number of contacts reported and age of participant, with the greatest number of contacts associated with 10–20-year olds and 40–50-year olds, and a sharp decline in contact rate above the age of 60 (figure 4*a*). We found no significant association of number of contacts and the sex of participants (electronic supplementary material, table S7). A greater number of contacts were associated with midweek days (Monday to Thursday than with weekend days (figure 4*b*, electronic supplementary material, table S7). The number of locations in which contacts were reported was associated with an increasing number of contacts (figure 4*b*, electronic supplementary material, table S7). Study wave 2 (R2) was associated with a significantly greater number of contacts than the other waves (electronic supplementary material, table S7).

We repeated the model fitting with number of contacts stratified by the social setting in which they were made (home, school or work, other) as independent models, to investigate the association between covariates and contact rates in different settings. We found the number of home contacts to be greatest in children and 40–45-year olds, and to increase with increasing household size (electronic supplementary material, table S7 and figure S7). We found no association of home contacts with week day or study wave. The greatest number of school or work contacts was associated with school- and working-age individuals, and contact number was greater for males than females and midweek days than weekends (electronic supplementary material, table S7 and figure S7). We found no association of number school/work contacts with contact locations greater than 1 or study wave. The number of contact made in other settings, which included leisure and shopping activities, was associated with age: the number of contacts in these settings decreased with age up to 30 years, and then increased with increasing age (electronic supplementary material, table S7 and figure S7). Females made more contacts in these settings than males, and more of these contacts were made at weekends; there was no effect of study wave. There was a very large effect of number of contact locations with these types of contact, suggesting that this type of contact may be responsible for the relationship with total number of contacts as the response variable. The number and proportion of contacts made in different social settings varied by study wave (electronic supplementary material, table S5).

Total contact duration was also significantly associated nonlinearly with participant age, with a general reduction in duration observed with increasing age (figure 4*c*). There was no significant effect of the sex of participants, but there was a significant effect of day of the week, with contact duration being longer on weekend days than Wednesdays Thursdays and Fridays (figure 4*d*, electronic supplementary material, table S7). Contact duration increased with the number of locations reported (figure 4*d*, electronic supplementary material, table S7). Study wave 2 was associated with shorter contact duration than the other waves (figure 4*d*, electronic supplementary material, table S6). We found our model findings to be insensitive to the uncertainty in the estimation of contact duration (electronic supplementary material, figure S8).

We found no significant association of number of locations visited with participant sex, study wave, but there was a significant nonlinear association with participant age, where 45–50-year olds were associated with the greatest number of locations visited, and more locations were reported on Fridays than other days of the week.

The questionnaire survey also asked participants whether the day for which they were reporting contact events could be considered as a ‘typical’ day or not. 73.4% of observations were reported to be typical days, 26.3% were reported as non-typical days and 0.03% (*n* = 13) of interviews participants could not be sure (they responded ‘Don't know’). Restricting our regression analysis to only observations categorized as ‘typical’ by participants gave similar associations with covariates as reported above (electronic supplementary material, figure S6).

### 3.5. Variation in contact rates

The longitudinal nature of our study and the random effect structure of our regression models allowed us to consider the proportion of variance in contact rate attributable to intra- and inter-individual variation (electronic supplementary material, table S8). When we considered the number of people encountered (the number of contacts), we found between individual variation (33.7%) to be less than the variation observed within individuals (66.3%). A similar distribution of variance was observed for total contact duration (28.6% between and 71.4% within individuals) and number of locations (25.9% between and 74.1 within individuals). When we limited our study observations to only those where participants reported ‘typical’ days, we found between variation to increase slightly, but still less than within individuals (electronic supplementary material, table S8). Similar patterns were found for models of number of contacts in different social settings (electronic supplementary material, table S8). Finally, to further explore wave-to-wave variation in individuals' contact rates, we considered how likely individuals were to report a consistent number of contacts across study waves, by calculating the percentage of participants remaining in the same contact quantile as the number of quantiles increased (electronic supplementary material, figure S10). Only between 30% and 40% of participants had consistent contact rates in the range of quantiles we explored, though we consistently found a greater proportion of participants’ observations remained in their quantile than for a null model which excluded within-participant dependencies.

### 3.6. Variation between individuals and neighbourhood saturation

As the number of observations per participant increases, reflecting a corresponding increase in infectious period, we may expect the variation in cumulative contacts between individuals to decrease. Subsequently, we hypothesize that infections with different infectious periods may inhabit potentially different dynamics networks of transmission opportunities, even in the same host population. We explored changes in the variation of cumulative contact rates over multiple study waves (electronic supplementary material, figure S11). While nearly all measures of between-participant variation decreased with increasing number of study waves, in many cases we found the variation to be greater than that expected by a null model which excludes within-participant dependencies. Thus, there was evidence that contact rates saturate to some extent (particularly for contact duration and number of locations), though within-individual variation is still sizable. We found evidence of weak positive correlation between an individual participant's coefficients of variation for number of contacts, contact duration and number of locations (electronic supplementary material, figure S12).

### 3.7. Individual and group contacts

To understand how our finding related to how participants reported contacts, we considered the number of contacts reported as individuals and as groups independently (electronic supplementary material, appendix H). Participants tended to report less than one group per diary on average, with an average group size of between 9 and 11 people (electronic supplementary material, table S9). The distribution of contact number reported as individuals was consistent across waves, though there was more variability between waves for contacts reported as groups (electronic supplementary material, figure S13). We also fitted independent regression models following the method of that described for the total number of contacts, with two different response variables: the number of contacts reported as individuals or groups (electronic supplementary material, figure S14). These models present similar results to the combined contact model, though associations deviate for several of the variables, most notably the relationship with number of locations and study wave. Whether a contact is reported as an individual or part of a group is the choice of the participant, and participants tended to use groups for reporting large number of contacts. These deviations from the principle model likely reflect differences between participants, and encounters between waves, and may also reflect participants tending to reporting groups more often for as they grew accustomed to participating in the study.

## 4. Discussion

Social encounter patterns are an important driver of the spread of infectious diseases requiring close contact for transmission, particularly for respiratory viral pathogens [3]. Quantifying such behaviours enables improved modelling of epidemics for a variety of purposes, and helps identify effective interventions aimed at reducing transmissions. Here, we present the results of a large longitudinal study of Hong Kong residents, a population inhabiting one of the highest density locations in the world and one which played an important role in the transmission of SARS [29].

We conducted a large cohort study where participants were asked to provide information on their social contacts and mixing behaviour at up to four different time points during two calendar years. At the aggregate level, we found remarkable consistency in the contact patterns made by participants across study waves, in terms of both the distribution of number and duration of contacts, as well as the distribution of number of distinct locations in which contacts were made and the pattern of mixing within and between different age groups. These aggregate contact patterns were similar to those observed in other studies based on European and Chinese populations [10,11,26,27,30], and a recent study of Hong Kong residents [31]. However, large representative studies of contact patterns have so far been limited to cross-sectional observations of behaviour over a single day per participant. Our findings suggest that this is an appropriate methodology when the objective of the study is to provide aggregate measures of contact patterns (e.g. contact rate per age group or age assortativity patterns) for modelling purposes. If the objective is to parametrize individual-based models or more finely resolved group-structured models; however, our findings suggest it may be important to incorporate individual-level variation in contact rate and social mixing behaviour.

We found a significant association of study wave on the number and duration of contact, and also found the pattern of mixing between age groups was subtly different between waves. We hypothesize that the effect of wave we observe may reflect seasonal patterns in contact behaviour, including Chinese New Year and summer school holidays. Differences in contact rate by wave may also be explained by the different ‘mix’ by wave of encounters made in different social settings. We found day of the week to be significantly associated with the number and total duration of contact, with weekends associated with fewer contacts but increased contact duration than weekdays.

The number of locations, the number of contacts and the time spent in contact with them appeared to be strongly linked. Individuals who visit more locations accrued a greater number of contacts, while contact duration quickly asymptotes with location number. Stratification by social settings in which encounters are made suggests that the effect of number of locations visits on contact number is driven by contacts made outside of the home, school and workplace environments. Individuals may visit many locations and make a correspondingly large number of contacts, but overall tend to spend less time per contact. This suggests an intriguing interplay between the spatial roaming of individuals and their network of social encounters in environments not easily represented by demographic and occupational-derived models, presenting a complex challenge in representing social or transmission networks within geographical space, and in developing realistic models of infectious disease transmission.

We found considerable intra-individual variation in contact rates reported by individuals, even after accounting for potential confounders (day of week, number of locations in which contact occurred and study wave): intra-individual variance was greater than inter-individual variation for the number and duration of contact and the number of locations in which contact occurred. While our study suggests that the number of contacts made by individuals is variable on a day-to-day basis, we find that the variation in contact rate reduces little when we consider the accumulation of contacts over multiple days. Our analysis is limited to only four observations per participant and our methodology does not permit unique contacts to be identified between waves. Nonetheless, these results suggest that inter-individual variation in the number or people encountered, the time spent in contact with them, and the variety of locations they are encountered in, may not saturate as quickly as expected over longer infectious periods. Our analysis does not explore the potential source of the intra- and inter-individual variation, and a deeper exploration of the setting and reasons for the contacts reported by participants may prove illuminating from both sociological modelling and public health perspectives.

Many respiratory pathogens of public health interest have infectious periods of longer than a single day, and the contacts made by infectious individuals during their period of infectivity will define the speed and extent of spread within the network. The re-wiring of an implicit contact network that we have measured may ensure that local saturation effects, where infectious individuals have opportunity to infect all susceptible individuals within their neighbourhood, are rare outside the household for pathogens with short infectious periods. The relationship of infectious period with the temporal dynamics and geographical patterns of social encounters we have observed is likely to drive the higher-order spread of infectious disease, and may provide important insights for public health interventions, such as contact tracing.

There are some limitations to this study. While our study is generally representative of Hong Kong households and population, we recruited very few participants under the age of 5 years old. We were also reliant on the recall of contact events by participants, and this might introduce bias in the number, duration and location of reported contacts. A further limitation is that as our study was conducted across several waves spanning several months, we do not have contact behaviour information from participants from consecutive days. A consequence of the telephone study team not working on weekends, and the random assignment of contact reporting days to participants meant that lower numbers of contact days were recorded for Saturdays and Sundays. The bias in sampling of different days of the week is, therefore, a consequence of our study design; the principal aim through sampling was to recruit a representative sample of households and individuals therein, and representativeness for day of the week was secondary in our sampling aims. Weather conditions may have a confounding effect on the contact patterns we have observed [32] and we did not adjust for these in our analysis. Finally, due to the design of data collection, we cannot identify repeated contact made between a participant and the same individual (their contact), which limits our ability to fully identify any neighbourhood saturation effect.

In conjunction with information from other studies, our study provides important information for the parameterization of realistic models of social encounters made in Hong Kong, with application to public health modelling. This study also provides support for the use of cross-sectional information for parameterizing epidemic models which focus on describing the risk of infection for average individuals. However, our study also highlights the complexity of social encounters, particularly when considering their spatial context, and the need for improved understanding of the social processes driving population-scale mixing patterns.

## Supplementary Material

Supplementary Materials

## Supplementary Material

Dataset

## Data accessibility

The dataset is available as electronic supplementary information.
