# Supplementary material for: Temporal variation of human encounters and the number of locations in which they occur: a longitudinal study of Hong Kong residents
Source: J R Soc Interface. 2018 Jan 24;15(138):20170838. doi: 10.1098/rsif.2017.0838 (PMC5805989; doi:10.1098/rsif.2017.0838)
Supplement: Supplementary Materials [file rsif20170838supp1.pdf]

Supplementary Material

List of Tables and Figures 2
Appendix A. Recording of contacts and locations.. 2
Appendix B. Study sample demography. 4
Appendix C. Comparison of contact patterns between study waves.................................. 4
Appendix D. Average number of total and touch contacts stratified by participant-contact age groups, setting, and study wave 10
Appendix E: Force of infection based on wave-specific age-mixing..................................13
Appendix F. Regression models: fixed and random effects, and sensitivity analyses. 14
Appendix G. Individual-level variation in contact rates...21
Appendix H. Contacts reported by participants as individual or group contacts. 24
Appendix I: Data Release 26

List of Tables and Figures

Table S1. Contact diary recording tables.
Table S2. Mean average of contact rates stratified by study wave.
Table S3. Comparison of individuals' contact metrics between different study waves.
Table S4. Mean number of contacts, stratified by study wave, and age group of participant and contact.
Table S5. Mean number of contacts, stratified by study wave, age group of participant, and social setting.
Table S6. Mean number of contacts involving touch, stratified by study wave, and age group of participant and contact.
Table S7. Estimated fixed effects of the regression models shown in Figure 4 of the main text, excluding spline terms.
Table S8. Variance within the random effects of the regression models.
Table S9. Mean and standard deviation of reported individual and group contacts, for each study wave.
Table S10. Data dictionary for released data.

Figure S1. Study population demography.
Figure S2a. Individual-level distribution of the different in number of contacts reported by participant between pairs of study waves.
Figure S2b. Individual-level distribution of the different in duration of contacts reported by participant between pairs of study waves.
Figure S2c. Individual-level distribution of the different in number of locations reported by participant between pairs of study waves.
Figure S3. Boxplots of total number of contacts made by age groups of participants stratified by study wave.
Figure S4. Correlations between age-mixing matrices from each study wave.
Figure S5. Force of infection estimates based on observed age-specific mixing patterns for each study wave.
Figure S6. Modelled contact rates for days reported by participants as being 'typical'.
Figure S7. Modelled number of contacts made in (A,B) home, (C,D) work or school, and (E,F) other social settings.
Figure S8. Sensitivity of fitted regression model to estimated contact durations.
Figure S9. Regression models exploring the relationship between the proportion of contacts involving touch and the average duration per contact and the total number of contacts reported.
Figure S10. Intra-participant variation in contact rates.
Figure S11. Plots examining how the coefficient of variation of different contact metrics changes as observations accumulate with each study wave.
Figure S12. Relationship between individual-level coefficient of variation (CoV) for number of contacts, contact duration and number of locations.
Figure S13. Distribution of number of contacts reported as individuals and group contacts, stratified by study wave.
Figure S14. Modelled number of contacts reported as (A,B) individuals and (C,D) groups.

Appendix A. Recording of contacts and locations.

A participant's contacts and characteristics of those contacts were recorded by study researchers through interviews with the participant, and the information provided was recorded by researchers in two tables shown in Table S1. Participants were interviewed and prompted to recall the people they encountered as well as *contact events*, where an event was defined as an encounter made with one or more other people in a particular location within a discrete time period, and an encounter was defined as a face-to-face conversation between a participant and another person where they are within 1 meter of each other and/or where a participant touched someone's

skin with their skin (examples provided to participants included shaking or holding hands, or a kiss). Each participant's contacts were assigned a unique name or description by the interviewer. Thus, a participant could record encounters made in the same location with possibly the same individuals but at very different times of the day (for example, meeting the same group of commuters on the way to work and on the way back from work).

Interviews proceeded as follows. First, subjects were asked to recall the different individuals or groups of individuals they encountered on the pre-assigned recording day, to populate the Person table (Table S1). Second, participants were asked to answer some basic information about each contact individual or group. Participants were asked to report: the age range of the contact(s) (0-4, 5-19, 20-39, 40-64, 65 or older); the typical frequency of encountering the contact(s) (Regular contact: 4 or more days a week, 2-3 days a week, once a week, Non-regular contact: less than once a week, met for the first time that day). For groups, participants were instructed to report the characteristics that would apply to the majority of individuals present within the group. Third, participants were asked to recall, for each individual/group encountered, the different locations in which they encountered that particular individual/group. Responses were used to populate the Contact Event Table (Table S1). Characteristics of the contact event were recorded at this time: whether the encounter involved skin-on-skin touch; the social context in which the contact event occurred (the participant's home, work or school, travel, shopping, meet or others), an estimate of the duration of the contact event (<10 minutes, 10-29 minutes, 30-59 minutes, 1 hour, 1 hour to 2 hours, 2 hours to 4 hours and 4 hours or more).

Table S1. Contact diary recording tables.

Table name	Items recorded
Person Table	• A short description of each individual or group of individuals encountered by the participant during their recording day (for interviewing purposes)<br>• Number of people encountered (if reporting a group)<br>• Age category of individual/group<br>• Typical frequency with which the participant encounters the individual/group
Contact Event Table	• Reference of individual/group encountered during contact event (link to entry in Person Table)<br>• Start time at which the participant was at that location (for reference purposes during interview)<br>• A short description of the location (for reference purposes during interview)<br>• Setting or setting of encounter event (home, work, school, shopping, restaurant, travel, leisure, other)<br>• Duration of the contact event<br>• Whether the contact event involved skin-on-skin touch

Appendix B. Study sample demography.

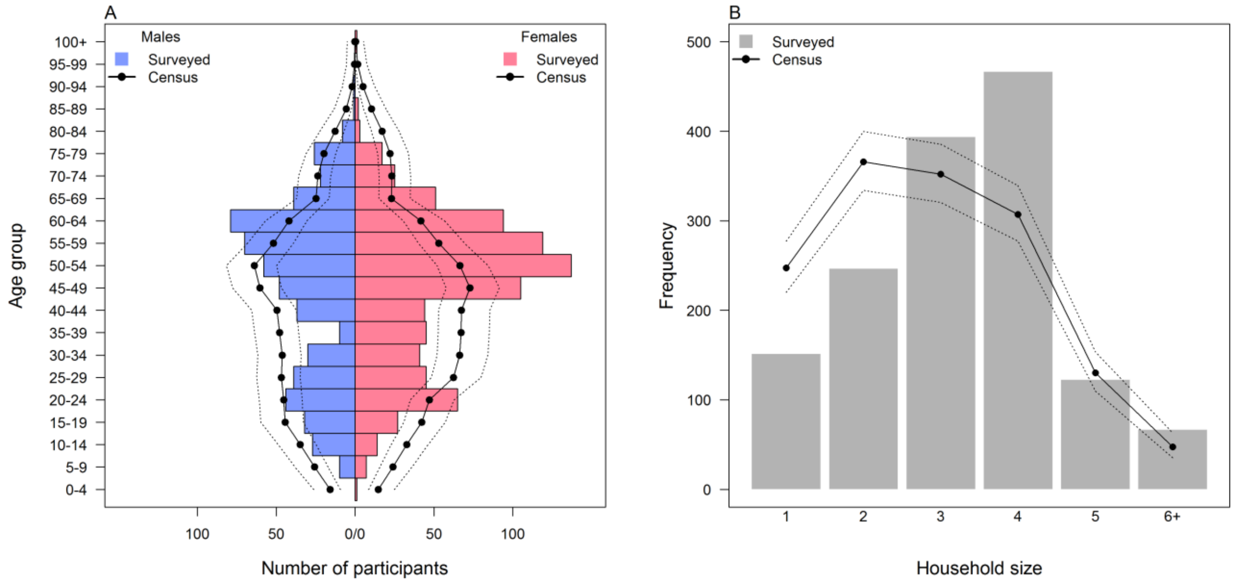

Figure S1. Study population demography. (A) Age and sex of participants at recruitment. (B) Household size of participant (including participant). Black dots denote expected distributions based on distributions derived from Census 2011 data provided by Hong Kong Census and Statistics Department. Dotted lines denote 95% confidence bound given final recruited participant across all waves.

Appendix C. Comparison of contact patterns between study waves.

Table S2. Mean average of contact rates stratified by study wave. Confidence intervals were derived from 1,000 bootstrap resamples of the data.

Contact metric	Study wave	Mean (95% confidence interval)
Number of contacts	R1	11.91 (10.75-13.11)
	R2	14.10 (12.21-16.08)
	R3	12.67 (11.23-14.32)
	R4	11.25 (9.71-13.08)
Total contact duration	R1	9.48 (8.88-10.08)
	R2	8.34 (7.80-8.84)
	R3	9.00 (8.42-9.62)
	R4	9.48 (8.82-10.12)
Number of locations	R1	2.98 (2.88-3.10)
	R2	2.96 (2.87-3.06)
	R3	3.01 (2.89-3.14)
	R4	3.00 (2.88-3.12)

Table S3. Comparison of individuals' contact metrics between different study waves. Confidence intervals were derived from 1,000 bootstrap resamples of the data.

Contact metric	Paired study waves	Mean difference (95% confidence interval)	Spearman's correlation coefficient
Number of contacts	R1-R2	-2.807 (-5.544, -0.208)	-0.022, p=0.556
	R1-R3	-0.742 (-3.185, 1.858)	-0.005, p=0.906
	R1-R4	0.325 (-2.298, 2.662)	-0.028, p=0.494
	R2-R3	1.693 (-1.483, 5.315)	-0.034, p=0.379
	R2-R4	2.187 (-0.706, 5.346)	-0.027, p=0.492
	R3-R4	1.800 (-1.389, 4.797)	0.060, p=0.132
Total contact duration	R1-R2	1.603 (0.570, 2.553)	-0.019, p=0.602
	R1-R3	0.460 (-0.783, 1.629)	-0.084, p=0.040
	R1-R4	0.235 (-0.985, 1.488)	0.014, p=0.727
	R2-R3	-0.996 (-1.926, -0.087)	0.080, p=0.042
	R2-R4	-1.069 (-1.977, -0.139)	0.032, p=0.422
	R3-R4	-0.227 (-1.280, 0.829)	-0.028, p=0.490
Number of locations	R1-R2	-0.048 (-0.209, 0.136)	0.035, p=0.336
	R1-R3	-0.073 (-0.272, 0.135)	-0.011, p=0.785
	R1-R4	-0.037 (-0.262, 0.168)	-0.026, p=0.525
	R2-R3	-0.062 (-0.243, 0.119)	-0.020, p=0.600
	R2-R4	-0.032 (-0.220, 0.178)	-0.008, p=0.829
	R3-R4	0.005 (-0.197, 0.216)	-0.002, p=0.966

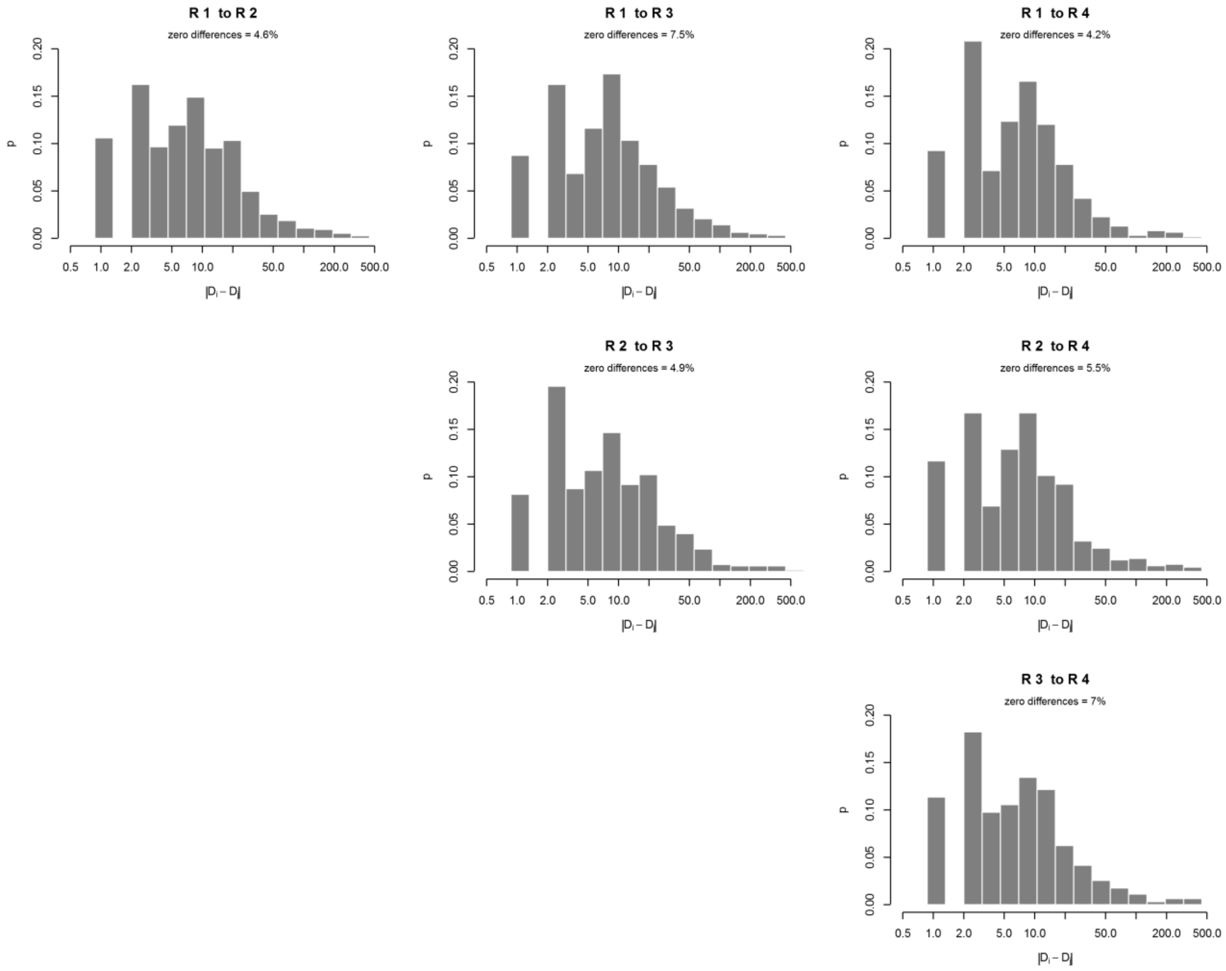

Figure S2a. Individual-level distribution of the different in number of contacts reported by participant between pairs of study waves. Note, the absolute difference is binned by logarithmically spaced breaks.

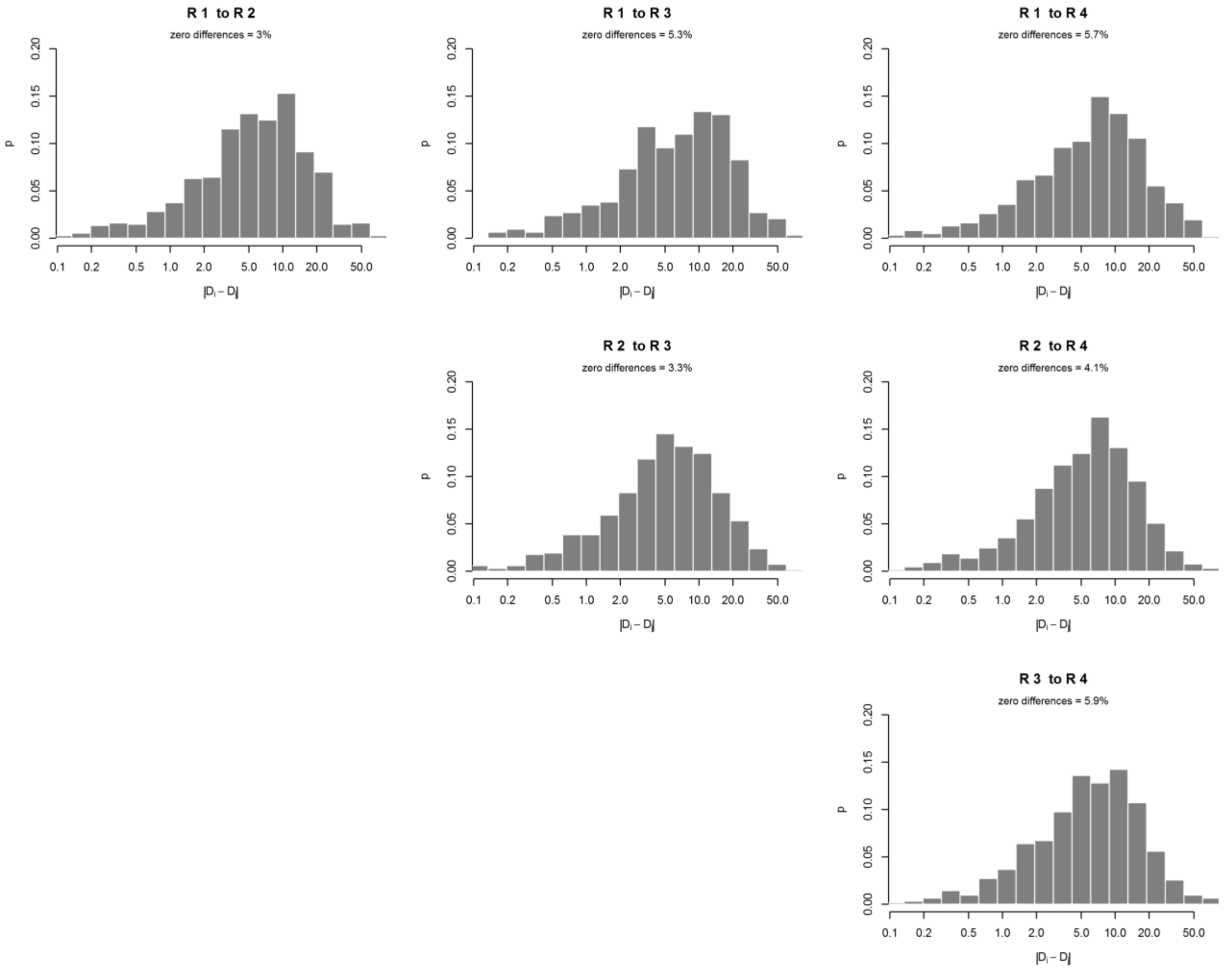

Figure S2b. Individual-level distribution of the different in duration of contacts reported by participant between pairs of study waves. Note, the absolute difference is binned by logarithmically spaced breaks.

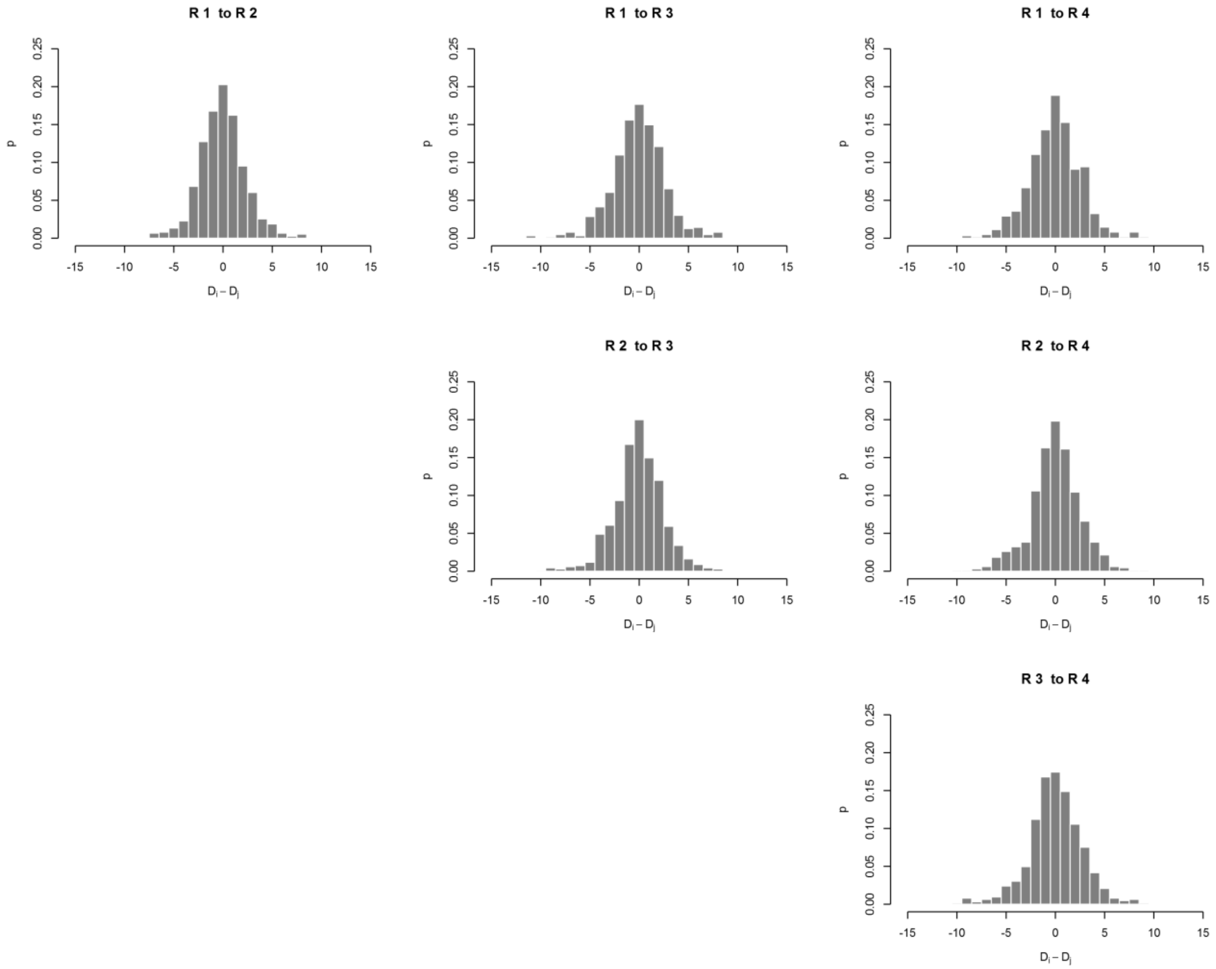

Figure S2c. Individual-level distribution of the different in number of locations reported by participant between pairs of study waves.

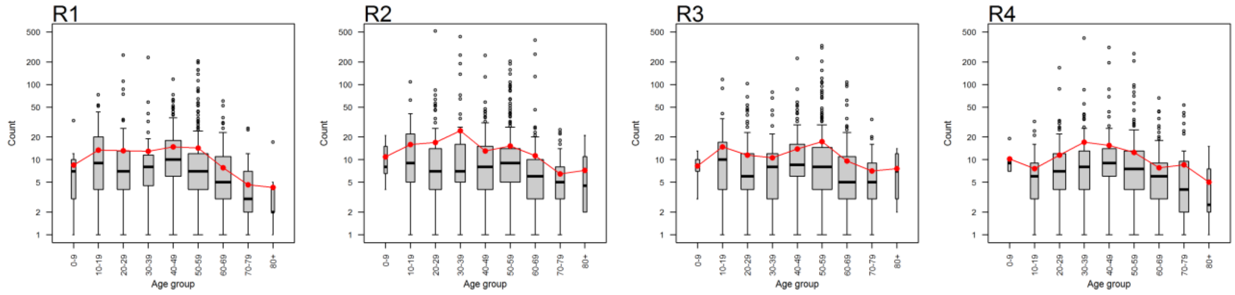

Figure S3. Boxplots of total number of contacts made by age groups of participants stratified by study wave. Box widths are indicative of the sample size. Red dots denote the mean number of contacts for each group. Note, the y-axis is plotted as a log-scale, and so participants making zero contacts are not represented. The number of zero contact observations in each study wave are as follows: R1, 21; R2 11; R3, 16; R4 21.

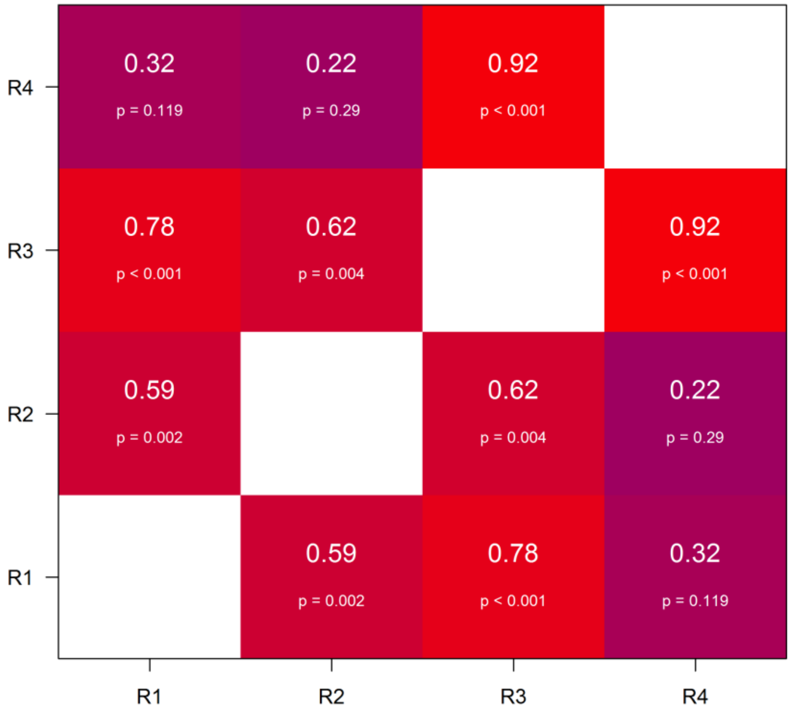

Figure S4. Correlations between age-mixing matrices from each study wave. This matrix shows the spearman correlation coefficients (and associated p-values) between pairs of wave-specific age-mixing matrices shown in Figure 4. R1 to R4 are each of the four study waves.

Appendix D. Average number of total and touch contacts stratified by participant-contact age groups, setting, and study wave

Table S4. Mean number of contacts, stratified by study wave, and age group of participant and contact.

		Age of participant[1]				
**Study wave**		**0-4**	**5-19**	**20-39**	**40-64**	**65+**
**R1**	*n*	*1*	*80*	*203*	*629*	*144*
	**0-4**	1.00	0.31	1.45	0.71	0.25
	**5-19**	1.00	8.53	3.27	3.05	0.31
**Age of contact**	**20-39**	4.00	3.15	8.99	6.12	1.48
	**40-64**	4.00	3.86	7.10	7.71	2.99
	**65+**	0.00	1.77	2.66	2.10	2.53
**R2**	*n*	*1*	*61*	*189*	*586*	*145*
	**0-4**	20.00	0.10	0.48	0.58	2.75
	**5-19**	0.00	10.69	5.83	3.30	2.73
**Age of contact**	**20-39**	0.00	2.54	14.53	6.47	4.56
	**40-64**	1.00	2.43	12.18	8.04	5.48
	**65+**	0.00	0.15	5.14	2.83	5.25
**R3**	*n*	*0*	*56*	*182*	*501*	*138*
	**0-4**	-	0.12	0.89	1.93	0.12
	**5-19**	-	10.30	3.24	3.91	0.49
**Age of contact**	**20-39**		1.89	6.52	7.11	2.46
	**40-64**	-	2.30	5.86	9.49	3.82
	**65+**	-	0.16	2.39	4.16	3.20
**R4**	*n*	*1*	*43*	*166*	*480*	*141*
	**0-4**	0.00	0.05	2.55	1.38	0.07
	**5-19**	6.00	3.72	4.55	3.20	0.23
**Age of contact**	**20-39**	0.00	3.16	9.60	6.82	2.13
	**40-64**	6.00	2.93	8.80	8.52	3.36
	**65+**	0.00	0.21	4.20	4.13	2.77

1. No adjustment for the age distribution of the population or participants has been made to mean contact numbers.
Diagonal (same age mixing) is marked for convenience

Table S5. Mean number of contacts, stratified by study wave, age group of participant, and social setting.

Study wave		All participants	Age of participant[1] 0-4	5-19	20-39	40-64	65+
**R1**	*n*	*1066*	*1*	*80*	*203*	*629*	*144*
**Setting**	**home**	2.56	4.00	3.77	2.54	2.61	1.74
	**school**	0.60	6.00	5.05	0.35	0.19	0.01
	**work**	4.82	0.00	1.75	7.48	5.42	0.33
	**other**	4.54	1.00	3.21	3.05	5.35	4.01
**R2**	*n*	*995*	*1*	*61*	*189*	*586*	*145*
**Setting**	**home**	2.53	1.00	3.74	2.69	2.41	2.28
	**school**	0.87	20.00	8.33	0.96	0.21	0.06
	**work**	6.33	0.00	0.07	13.41	6.25	0.52
	**other**	4.78	0.00	3.90	3.19	4.95	6.79
**R3**	*n*	*887*	*0*	*56*	*182*	*501*	*138*
**Setting**	**home**	2.17	-	2.73	2.35	2.21	1.60
	**school**	0.67	-	7.27	0.42	0.05	0.51
	**work**	5.37	-	0.00	5.65	7.16	0.86
	**other**	4.89	-	4.21	3.04	5.74	4.48
**R4**	*n*	*836*	*1*	*43*	*166*	*480*	*141*
**Setting**	**home**	2.16	2.00	2.42	2.67	2.20	1.40
	**school**	0.23	0.00	1.53	0.29	0.16	0.00
	**work**	4.27	0.00	0.35	8.76	4.13	0.67
	**other**	5.00	7.00	4.21	2.57	5.94	5.01

1. No adjustment for the age distribution of the population or participants has been made to mean contact numbers.

Table S6. Mean number of contacts involving touch, stratified by study wave, and age group of participant and contact.

		Age of participant[1]				
**Study wave**		**0-4**	**5-19**	**20-39**	**40-64**	**65+**
**R1**	*n*	*1*	*80*	*203*	*629*	*144*
	**0-4**	1.00	0.06	0.25	0.22	0.09
	**5-19**	1.00	3.23	0.49	1.31	0.06
**Age of contact**	**20-39**	4.00	1.27	1.75	1.29	0.28
	**40-64**	3.00	1.69	1.50	1.68	0.83
	**65+**	0.00	0.16	0.38	0.41	0.61
**R2**	*n*	*1*	*61*	*189*	*586*	*145*
	**0-4**	0.00	0.03	0.30	0.27	0.16
	**5-19**	0.00	6.03	0.71	1.12	0.24
**Age of contact**	**20-39**	0.00	0.77	1.83	0.96	2.06
	**40-64**	1.00	1.00	1.14	1.12	0.97
	**65+**	0.00	0.07	0.35	0.43	0.72
**R3**	*n*	*0*	*56*	*182*	*501*	*138*
	**0-4**	-	0.04	0.14	0.46	0.10
	**5-19**	-	5.50	0.60	1.01	0.09
**Age of contact**	**20-39**	-	0.64	1.40	0.68	0.26
	**40-64**	-	1.27	1.06	1.27	0.54
	**65+**	-	0.07	0.46	0.61	0.34
**R4**	*n*	*1*	*43*	*166*	*480*	*141*
	**0-4**	0.00	0.05	0.13	0.15	0.07
	**5-19**	2.00	1.86	0.70	0.48	0.10
**Age of contact**	**20-39**	0.00	1.02	2.08	1.15	0.26
	**40-64**	5.00	1.44	1.23	1.89	0.33
	**65+**	0.00	0.07	0.22	0.59	0.64

1. No adjustment for the age distribution of the population or participants has been made to mean contact numbers.
Diagonal (same age mixing) is marked for convenience.

Appendix E: Force of infection based on wave-specific age-mixing.

Here we estimate the Force of infection (λ) for each study wave using the observed age-mixing patterns as shown in Figure 3 of the main text. We assume that the population is entirely susceptible, except for initial infecteds, and that the transmission rate per contact is constant and independent of participant or contact age. The population size in each age class is N_i, based on Hong Kong Census information. We make two assumptions regarding the number of initial infectious individuals in each age class I_i: (1) the number in each age category within the population is a fixed proportion, $I_i = pN_i$; (2) the number in each age category is the same across all age categories, $I_i = \frac{N_i}{\sum N_i} \times \sum pN_i$, such that the total number of infectious individuals under each assumption is the same.

We define the force of infection for each age class as

$$\lambda_i = \sum_j \beta Q_{ij} I_j$$

where Q_{ij} is the observed average number of contacts made by age class i with age class j per day, and β is the transmission rate per day given contact.

We present the age-class specific force of infection by study wave in figures **S5A** and **S5B** below and the total Force of Infection ($\sum \lambda_i$) by wave for each assumption regarding initial infected in figure **S5C**. We used $\beta = 1e^{-4}$, and $p = 1e^{-4}$, which corresponds to 697 initial infecteds in a population of 6,971,882. We excluded the 0 to 4 age class from our force of infection calculations (though did include this age group as potential infectors) due to the low number of observations in our study for these ages.

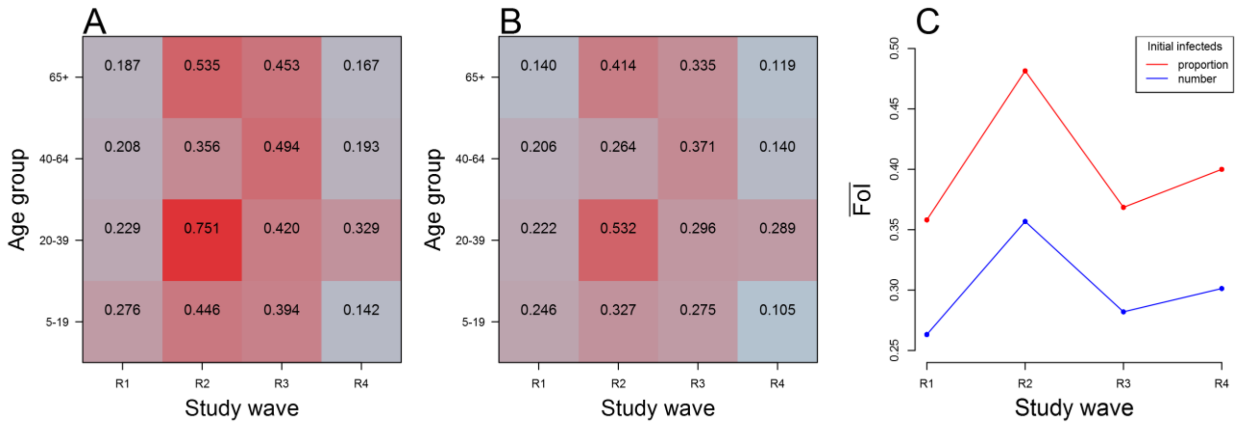

Figure S5. Force of infection estimates based on observed age-specific mixing patterns for each study wave. **A**ge-pair specific force of infection estimates assuming the same proportion (A) or number (B) of infecteds in each age group. (C) Average force of infection across age-groups, weighted by census-derived population size of each age group, over each study wave.

Appendix F. Regression models: fixed and random effects, and sensitivity analyses.

Table S7. Estimated fixed effects of the regression models shown in Figure 4 of the main text, excluding spline terms.

		**Model outcome**								
		**Number of contacts**			**Duration of contact**			**Number of locations**		
**Variable**		Estimate	Standard Error	P value	Estimate	Standard Error	P value	Estimate	Standard Error	P value
Intercept		1.219	0.049	<0.001	1.621	0.056	<0.001	1.293	0.023	<0.001
Sex	Male	0	-	-	0	-	-	0	-	-
	Female	0.027	0.032	0.393	0.013	0.035	0.711	0.020	0.017	0.233
Day of week	Saturday	0	-	-	0	-	-	0	-	-
	Sunday	-0.015	0.041	0.719	-0.005	0.048	0.912	-0.014	0.024	0.555
	Monday	0.096	0.041	0.019	-0.080	0.047	0.089	0.010	0.023	0.669
	Tuesday	0.108	0.042	0.009	-0.064	0.048	0.182	0.015	0.024	0.518
	Wednesday	0.135	0.044	0.002	-0.184	0.051	<0.001	0.019	0.025	0.459
	Thursday	0.122	0.043	0.004	-0.241	0.050	<0.001	0.003	0.024	0.915
	Friday	0.073	0.044	0.096	-0.132	0.050	0.009	0.089	0.025	<0.001
Number of locations	0	0	-	-	0	-	-	-	-	-
	1	0.679	0.036	<0.001	0.380	0.042	<0.001	-	-	-
	2	0.882	0.037	<0.001	0.528	0.043	<0.001	-	-	-
	3	1.123	0.042	<0.001	0.607	0.048	<0.001	-	-	-
	4	1.290	0.051	<0.001	0.695	0.059	<0.001	-	-	-
	5	1.320	0.062	<0.001	0.749	0.071	<0.001	-	-	-
	6 or more	1.446	0.063	<0.001	0.884	0.072	<0.001	-	-	-
Study wave	R1	0	-	-	0	-	-	0	-	-
	R2	0.089	0.029	0.002	-0.085	0.034	0.012	-0.003	0.017	0.857
	R3	0.056	0.030	0.062	-0.049	0.035	0.162	0.002	0.017	0.917
	R4	0.009	0.030	0.755	0.018	0.036	0.617	0.005	0.018	0.778

(Table S7 continued on following page)

Table S7 continued.

		Model outcome								
		**Number of contacts Home**			**Number of contacts School or work**			**Number of contacts Other**		
**Variable**		Estimate	Standard Error	P value	Estimate	Standard Error	P value	Estimate	Standard Error	P value
Intercept		0.851	0.044	<0.001	0.013	0.078	0.864	0.220	0.053	<0.001
Sex	Male	0	-	-	0	-	-	0	-	-
	Female	-0.006	0.022	0.804	-0.162	0.049	0.001	0.129	0.030	<0.001
Day of week	Saturday	0	-	-	0	-	-	0	-	-
	Sunday	-0.054	0.032	0.089	-0.084	0.065	0.199	0.148	0.045	0.001
	Monday	-0.083	0.031	0.008	0.486	0.065	<0.001	-0.158	0.045	<0.001
	Tuesday	-0.095	0.032	0.003	0.492	0.066	<0.001	-0.133	0.046	0.004
	Wednesday	-0.109	0.034	0.001	0.564	0.070	<0.001	-0.164	0.049	0.001
	Thursday	-0.139	0.033	<0.001	0.613	0.068	<0.001	-0.177	0.047	<0.001
	Friday	-0.096	0.033	0.004	0.533	0.069	<0.001	-0.159	0.048	0.001
Household size	1	0	-	-	-	-	-	-	-	-
	2	0.095	0.040	0.018	-	-	-	-	-	-
	3	0.295	0.038	<0.001	-	-	-	-	-	-
	4	0.445	0.039	<0.001	-	-	-	-	-	-
	5	0.541	0.049	<0.001	-	-	-	-	-	-
	6+	0.585	0.064	<0.001	-	-	-	-	-	-
Number of locations	0	-	-	-	0	-	-	0	-	-
	1	-	-	-	0.501	0.057	<0.001	0.586	0.040	<0.001
	2	-	-	-	0.500	0.059	<0.001	1.125	0.041	<0.001
	3	-	-	-	0.603	0.066	<0.001	1.506	0.045	<0.001
	4	-	-	-	0.566	0.082	<0.001	1.865	0.056	<0.001
	5	-	-	-	0.565	0.098	<0.001	1.887	0.068	<0.001
	6 or more	-	-	-	0.449	0.099	<0.001	2.169	0.069	<0.001
Study wave	R1	0	-	-	0	-	-	0	-	-
	R2	0.009	0.023	0.682	0.120	0.046	0.009	0.020	0.033	0.542
	R3	-0.064	0.023	0.006	0.073	0.048	0.127	0.060	0.034	0.079
	R4	-0.052	0.024	0.030	-0.007	0.049	0.889	0.059	0.035	0.090

Table S8. Variance within the random effects of the regression models. This table contains the variance associated with the random effect terms of the models presented in Figure 4 of the main text (Section A, using all observations), and those from additional regression models which restricted the observations to those from participants reporting their contact day was a 'typical' day (section B) or where the outcome was the number of contacts reported in a specific setting (section C).

Data	Outcome variable	Individual variation	Variance	% variance
**A**<br>All observations<br>(no. obs. = 3,383;<br>n. parts. = 1,123)	Number of contacts	inter	0.14622	33.7
		intra	0.28756	66.3
	Duration of contacts	inter	0.16054	28.6
		intra	0.40113	71.4
	Number of locations	inter	0.03523	25.9
		intra	0.10069	74.1
**B**<br>Only days reported by participants as 'typical'<br>(n. obs.= 2,016;<br>n. parts. = 740)	Number of contacts	inter	0.18080	42.3
		intra	0.24676	57.7
	Duration of contacts	inter	0.17078	30.7
		intra	0.38556	69.3
	Number of locations	inter	0.03969	34.0
		intra	0.07711	66.0
**C**<br>All observations<br>(no. obs. = 3,382;<br>n. parts. = 1,123)	Number of contacts made in home settings	inter	0.05309	22.0
		intra	0.18820	78.0
	Number of contacts made in school or work settings	inter	0.31160	29.3
		intra	0.75138	70.7
	Number of contacts not made in home, school or work settings	inter	0.07372	14.9
		intra	0.42127	85.1

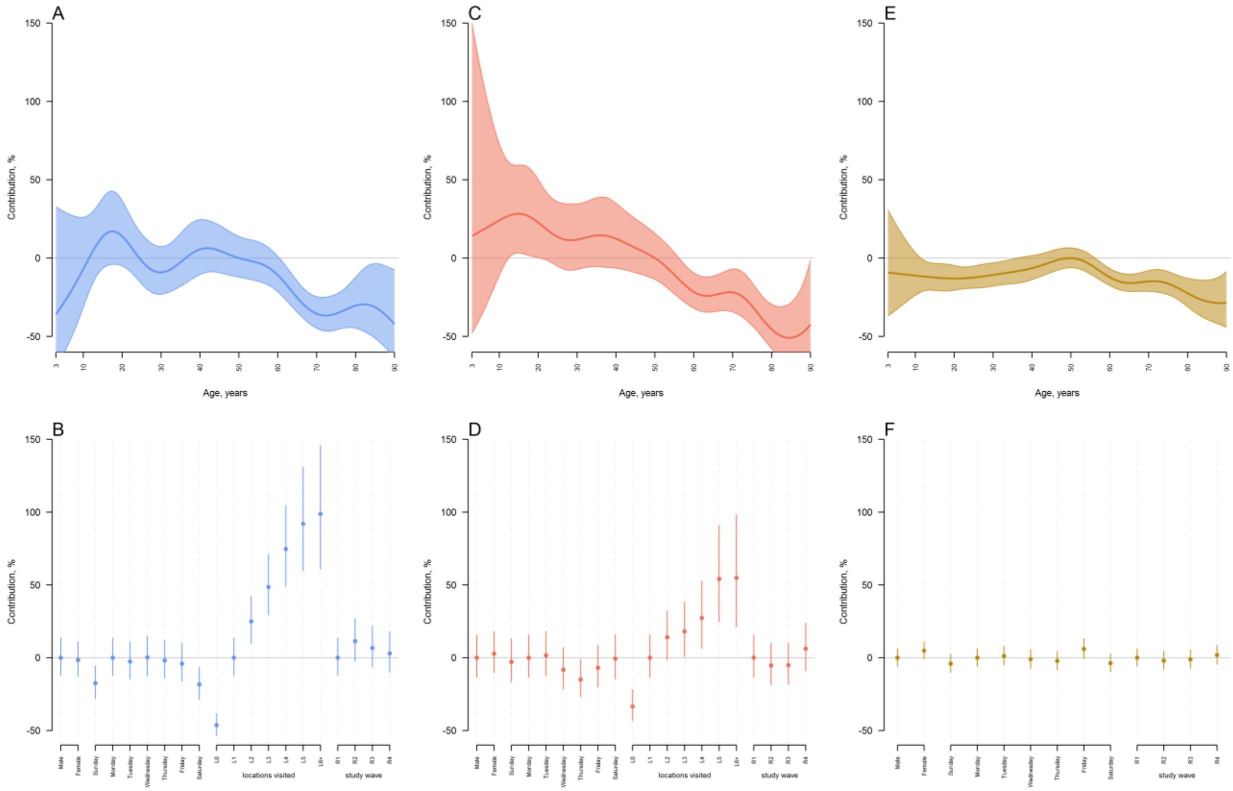

Figure S6. Modelled contact rates for days reported by participants as being 'typical'. Here we show the percentage contribution to contact rate (number of contact, duration of contact, number of locations) by the various covariates included in each model, relative to the contact rate predicted for a male 50-year-old from a household of size 1, on a Monday, with one contact location and during study wave 1. Models were fitted to data restricted to observations where participants reported their reporting day to be 'typical' and participants for whom there were at least two observations. Outcome variables were number of contacts (A and B), the total duration of contact events (C and D), and the number of locations in which contact occurred (E and F).

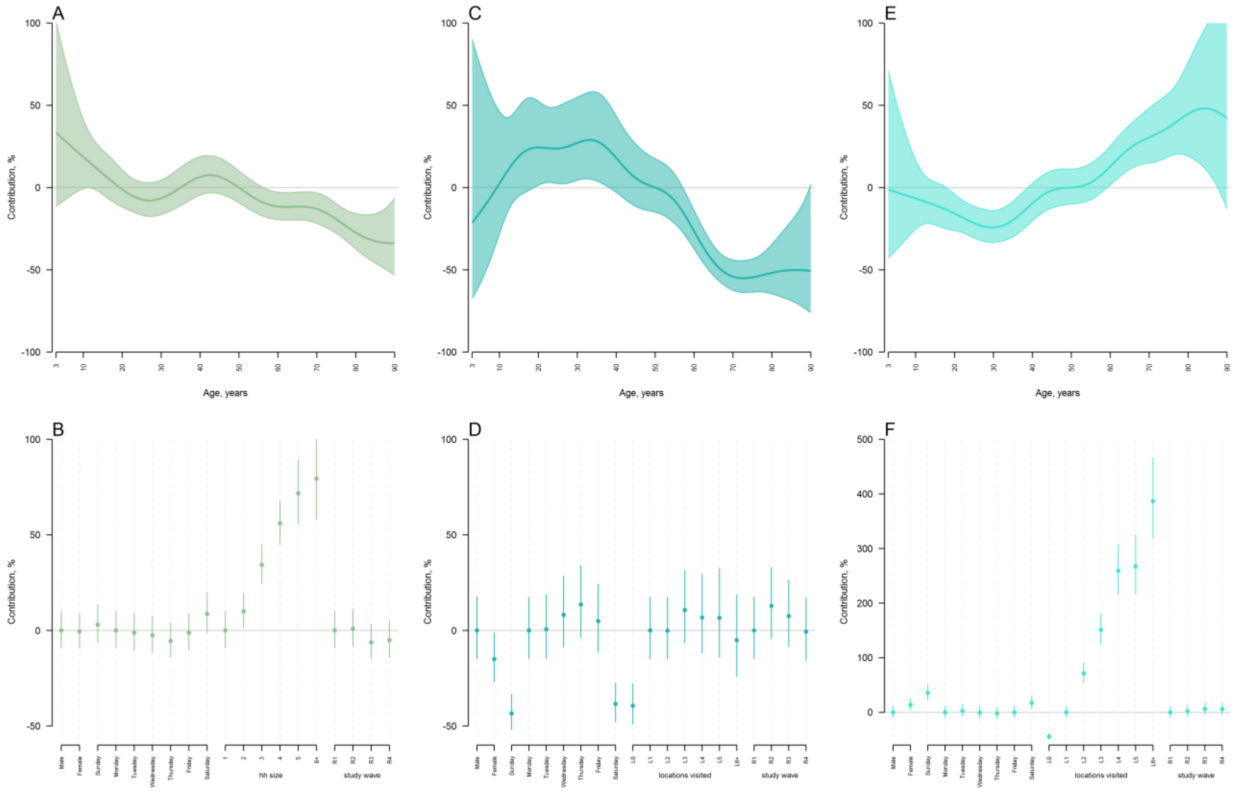

Figure S7. Modelled number of contact made in (A,B) home, (C,D) work or school, and (E,F) other social settings. Here, we show the percentage contribution to the number of contacts by covariates included in each model, relative to the contact rate predicted for a male 50-year-old from a household of size 1, on a Monday, with one contact location and during study wave 1. Models were fitted to data restricted to observations where participants for whom there were at least two observations. Note, for the number of contacts at home model (A,B), we excluded number of locations as an explanatory variable and instead included household size as a variable.

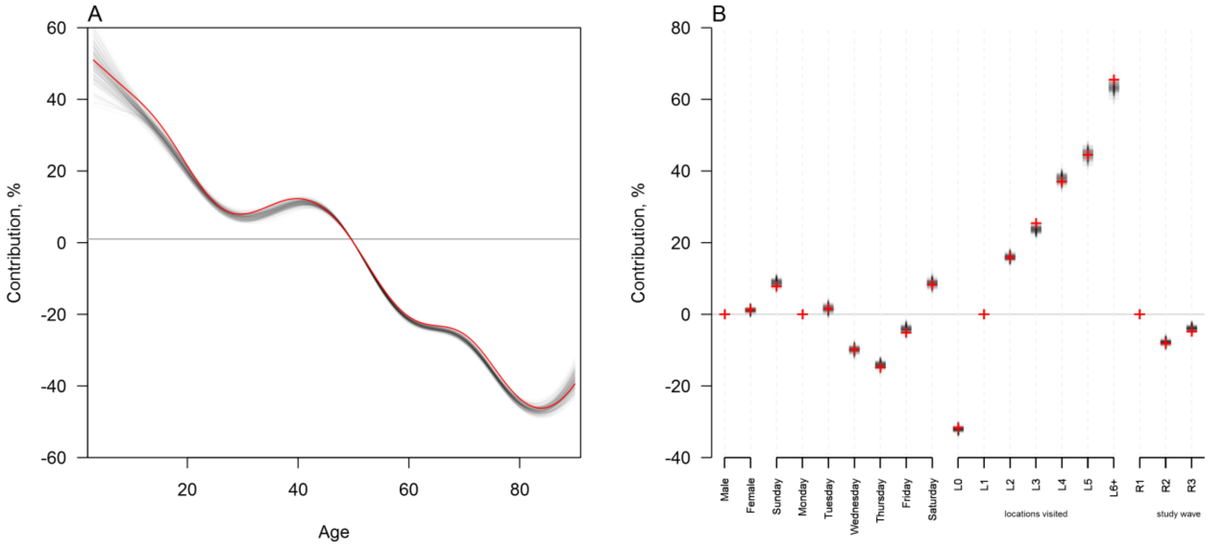

Figure S8. Sensitivity of fitted regression model to estimated contact durations. 200 estimates of contact duration were calculated for each contact event reported (with a valid contact duration category), and consequently there are 200 estimates of the total contact duration for each observation (participant-wave). Here, we explore the sensitivity of the regression model fit presented in Figure 4b/4d and Table S3 by fitting the same regression model to each set of 200 observations independently. (A) Predicted age contribution curves for all 200 models (grey lines) and the model reported in the paper (red line). (B) Predicted contribution for the other fixed effects of the 200 models (black crosses) and the model reported in the paper (red crosses).

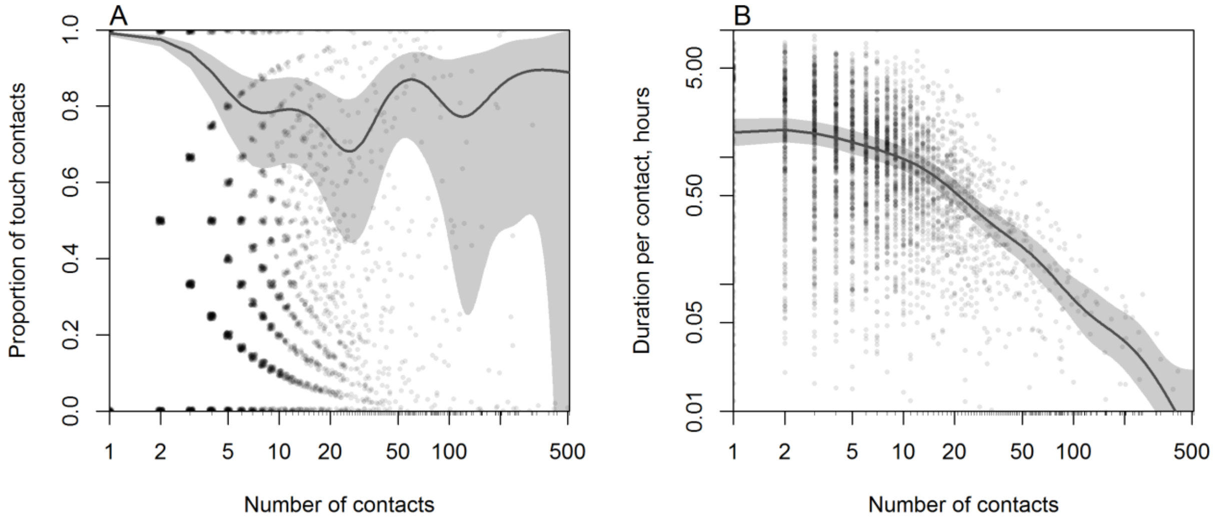

Figure S9. Regression models exploring the relationship between the proportion of contacts involving touch and the average duration per contact and the total number of contacts reported. The plots show the splines fitted to logged number of contacts as an explanatory variable, with (**A**) proportion of touch contacts and (**B**) the log

average duration per contact as response variables. Models adjusted for age (spline), sex, day of the week, household size, and study. Raw observations are shown as points in both plots, though they are jittered in **A** for clarity.

Appendix G. Individual-level variation in contact rates.

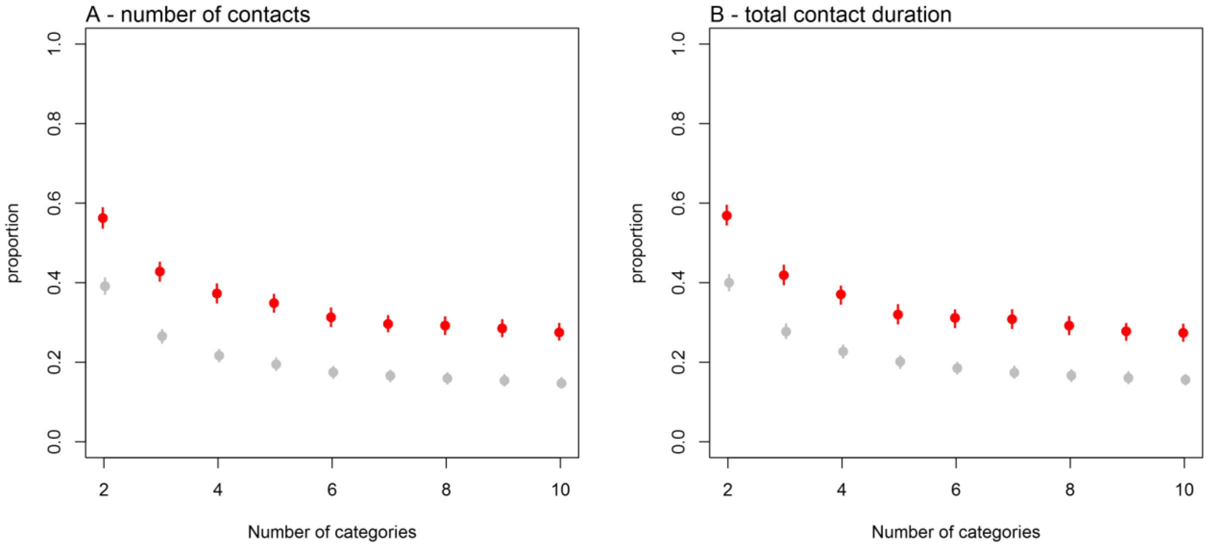

Figure S10. Intra-participant variation in contact rates. Proportion of individuals (with two or more observations) who remain within a single contact rate quantile category across all waves, against the number of quantiles used, for (A) number of contacts and (B) contact duration. Bootstrap estimates for both observed data (red) and null model synthetic data (grey) are shown. Null 'synthetic' data was generated from our observed data, where the individual-level contact metrics for study wave are resampled without replacement from the observations – essentially breaking the within-individual dependencies of our observed contact rates, while preserving the distribution of rates within each wave. Here, we assign each participant's wave-specific contact rate into a quantile category. Category breaks were defined by finding the required number of quantiles from all observed contact rates for individuals participating for their first time. We excluded individuals for which there was only a single (wave) observation. Lines represent bootstrapped 95% confidence intervals, which were generated through 500 resamples.

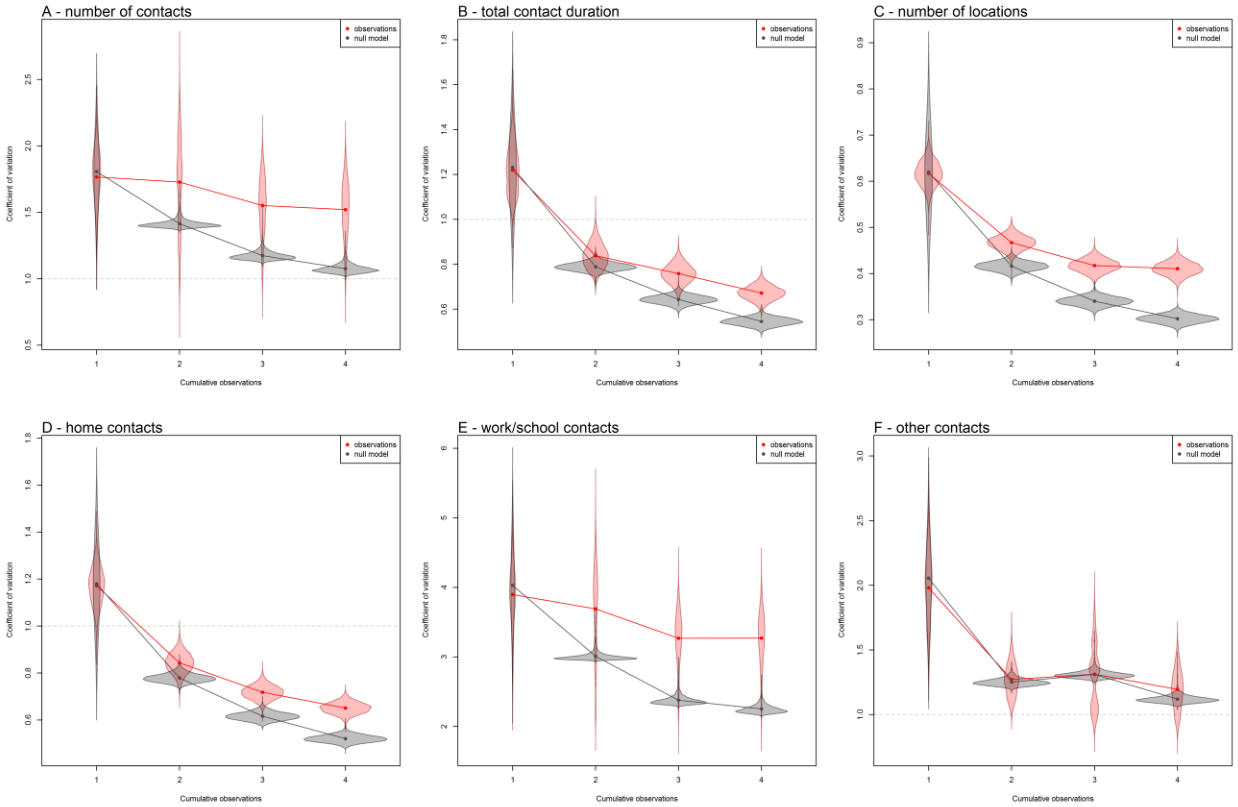

Figure S11. Plots examining how the coefficient of variation of different contact metrics changes as observations accumulate with each study wave. Contact metrics are (A) number of contacts, (B) total contact duration, (C) number of locations in which contact was made, (D) number of contacts made in home setting, (E) number of contacts made in school or work settings, (F) number of contacts made in other settings. Only individuals who participated in all four waves were considered (*n*=401). For each level of cumulative observations and for each individual, we calculate the cumulative contact metric reported (total number of contacts, total duration or total number of locations). We then calculate the coefficient of variation for that population of individuals. Pale red regions represent the distributions of CoV derived from 5,000 bootstrap resamples of the 401 participants. Grey regions show the equivalent CoV distributions for observation based synthetic data, where the individual-level contact metrics for study wave are resampled without replacement from the observations – essentially breaking the within-individual dependencies of our observed contact rates, while preserving the distribution of rates within each wave – and cumulative metrics derived.

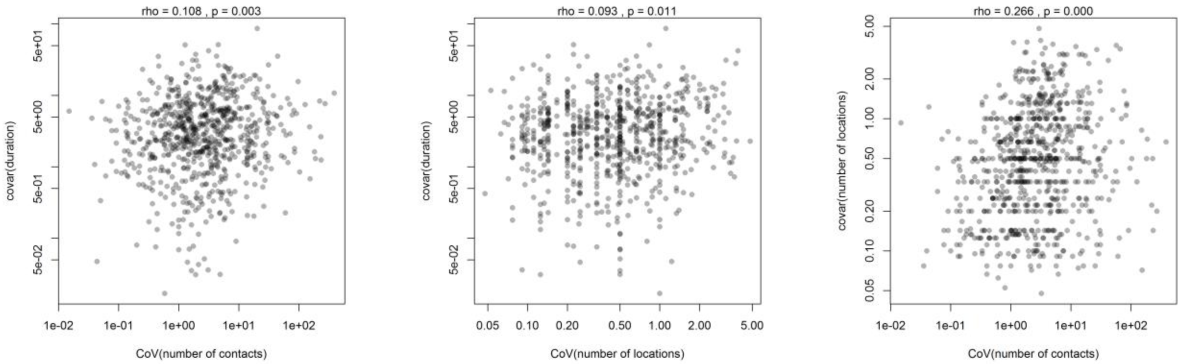

Figure S12. Relationship between individual-level coefficient of variation (CoV) for number of contacts, contact duration and number of locations. Spearman correlation estimates and associated p-values are shown above their corresponding plot. Only participants with 3 or more observations are included (number of participants=803).

Appendix H. Contacts reported by participants as individual or group contacts.

Table S9. Mean and standard deviation of reported individual and group contacts, for each study wave.

	**Study wave[1]**			
	**R1**	**R2**	**R3**	**R4**
Combined contacts	11.91 (20.34)	14.10 (32.76)	12.67 (23.57)	11.25 (24.62)
Individual contacts	5.25 (4.47)	4.34 (3.39)	4.62 (3.81)	4.50 (3.58)
Group contacts	6.66 (20.18)	9.76 (32.90)	8.05 (23.52)	6.74 (24.51)
Number of groups	0.61 (1.08)	0.93 (1.32)	0.82 (1.28)	0.83 (1.20)
Group size	11.34 (18.67)	10.22 (23.03)	9.46 (14.88)	8.72 (22.70)
Number of locations	2.98 (1.71)	2.96 (1.64)	3.01 (1.86)	3.00 (1.86)

1. mean (standard deviation)

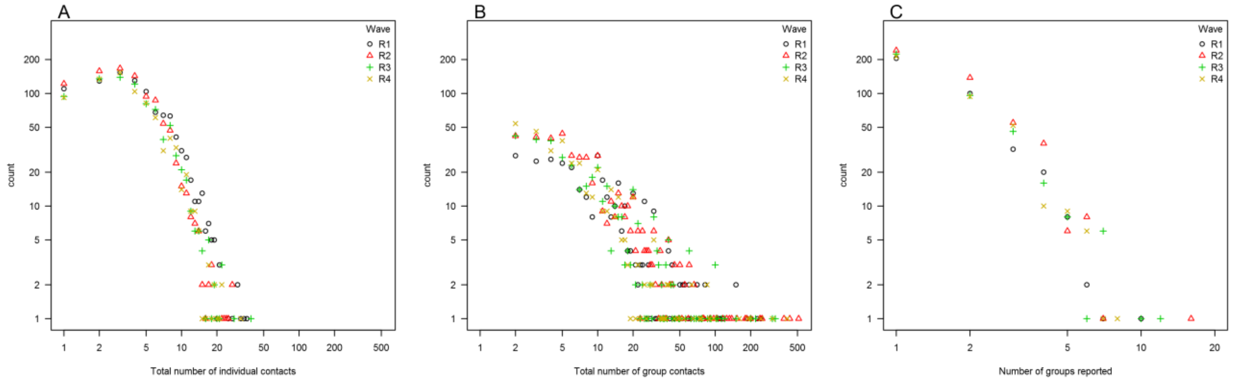

Figure S13. Distribution of number of contacts reported as individuals and group contacts, stratified by study wave. Distributions for (A) individual contacts, (B) group contacts, and (C) Distribution of the number of groups reported by participants, stratified by study wave.

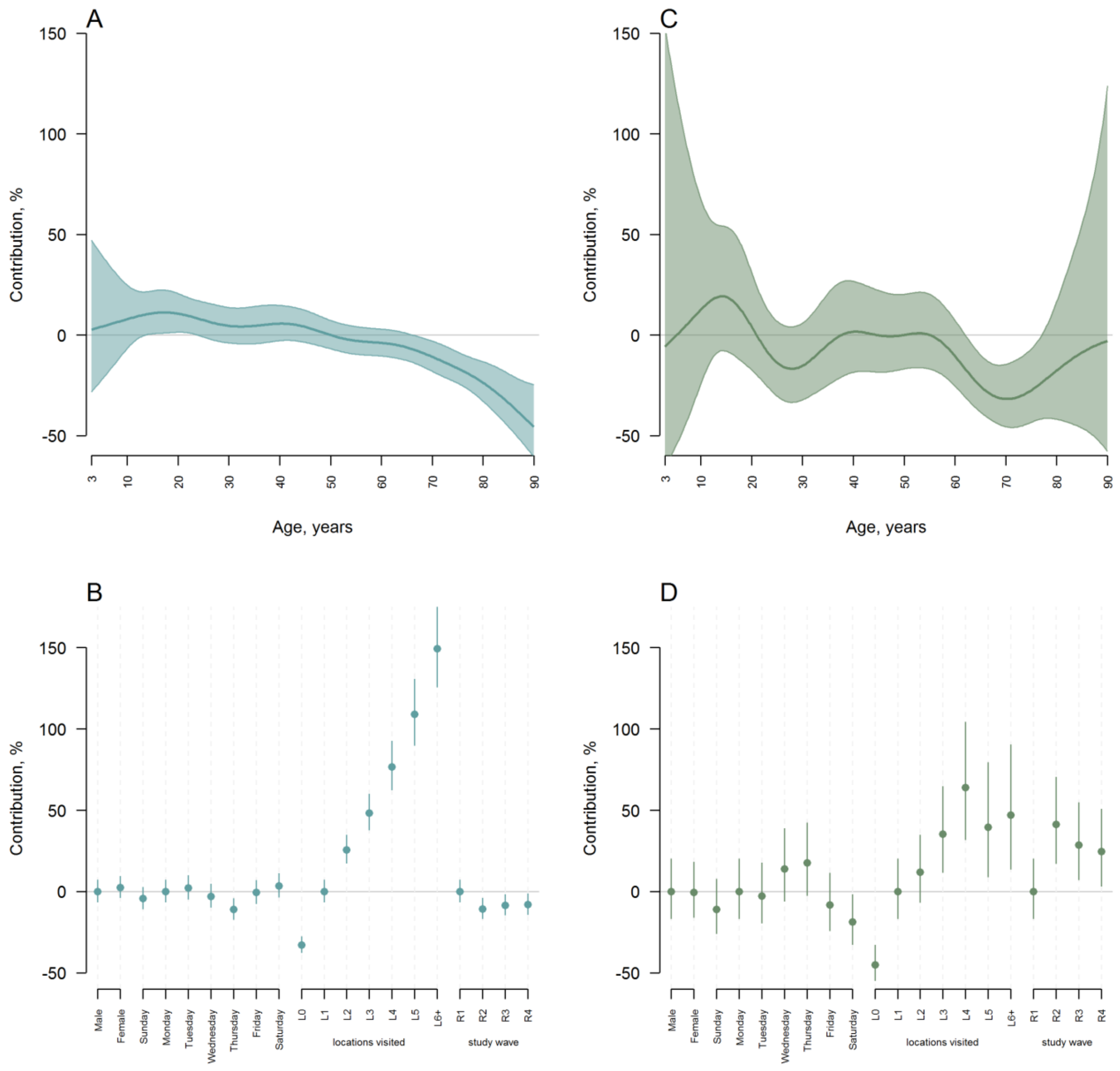

Figure S14. Modelled number of contacts reported as (A,B) individuals and (C,D) groups. Here, we show the percentage contribution to the number of contacts by covariates included in each model, relative to the contact rate predicted for a male 50-year-old from a household of size 1, on a Monday, with one contact location and during study wave 1. Regression analysis performed as for the main text, apart from the new outcome variables.

Appendix I: Data Release

Two data files are released with this manuscript: **hk_contact_number.csv** and **hk_contact_duration.csv**.

- **hk_contact_number.csv** – Information for each participant observation (a single recording day within a wave) including participant information, total number of contacts and number of locations. Rows = 3784, columns = 26. A data dictionary is provided in Table S10.
- **hk_contact_duration.csv** – 200 estimates of the total duration (minutes) of contact events, corresponding to the observations in **hk_contact_number.csv**. Rows = 3784, columns = 200. Column 1 was used as the outcome variable for the regression models presented in the main text and ESM. The value of theta used in the exponential model to estimate the contact durations was 0.01355328, and was fitted using an adaptation of the expectation–maximization algorithm as described in Read, J.M., et al., Social mixing patterns in rural and urban areas of southern China. *Proc Biol Sci*, 2014. 281(1785): p. 20140268.

Table S10. Data dictionary for released data.

Variable name	Description	Type
pid	Participant ID code.	Integer
hid	Household ID code.	Integer
age	Age (years) of the participant on the day of the observation. Participants older than 85 are assigned an age of 85 to preserve anonymity.	Integer
sex	Sex of the participant.	Categorical
n.samples	Number of observations (in total) for this individual	Integer
wave	Study wave, corresponding to R1 – R4.	Integer
reporting.day	Day of the week for which contact was reported.	Categorical
typical.day	Was this a typical day? (yes, no)	Categorical
n.contact.total	Total number of contacts reported. Specifically, the total number of unique individuals as identified through unique person/group descriptors and number of individuals reported (if a group).	Integer
n.locations	Number of unique contact locations reported	Integer
n.loc.group	Number of unique contact locations reported: 0, 1, 2, 3, 4, 5, 6+.	Categorical
n.contact.0-4	Number of contacts reported where contacts were between 0 and 4 years old.	Integer
n.contact.5-19	Number of contacts reported where contacts were between 5 and 19 years old.	Integer
n.contact.20-39	Number of contacts reported where contacts were between 20 and 39 years old.	Integer
n.contact.40-64	Number of contacts reported where contacts were between 40 and 64 years old.	Integer
n.contact.65+	Number of contacts reported where contacts were 65 years old or older.	Integer
n.contact.touch	Number of contacts involving touch	integer
n.contact.0-4.touch	Number of contacts involving touch where contacts were between 0 and 4 years old.	Integer
n.contact.5-19 touch	Number of contacts involving touch where contacts were between 5 and 19 years old.	Integer
n.contact.20-39 touch	Number of contacts involving touch where contacts were between 20 and 39 years old.	Integer
n.contact.40-64 touch	Number of contacts involving touch where contacts were between 40 and 64 years old.	Integer
n.contact.65+ touch	Number of contacts involving touch where contacts were 65 years old or older.	Integer
n.contact.home	Number of contacts made within a home setting.	Integer
n.contact.school	Number of contacts made within a school setting.	Integer
n.contact.work	Number of contacts made within a workplace setting.	Integer
n.contact.other	Number of contacts made within any other setting.	Integer
